# Strategies toward efficient tin-based perovskite solar cells

**DOI:** 10.1039/d6sc06089g

**Published:** 2026-09-07

**Authors:** Tomoya Nakamura, Chien-Yu Chen, Atsushi Wakamiya

**Affiliations:** a Institute for Chemical Research, Kyoto University Gokasho Uji Kyoto 611-0011 Japan tomoya@scl.kyoto-u.ac.jp cychen@scl.kyoto-u.ac.jp wakamiya@scl.kyoto-u.ac.jp; b HIKARI-COOL Kyoto, Kyoto University Institute for Advanced Study Yoshida Ushinomiya-cho Sakyo-ku Kyoto 606-8501 Japan

## Abstract

Tin halide perovskite solar cells are considered one of the most promising alternatives to conventional lead-based perovskite photovoltaics due to their structural and electronic similarities, along with their lower toxicity. However, the chemistry of tin halide perovskites is distinct from that of their lead counterparts. As a result, tin halide perovskite solar cells still lag behind lead-based devices in both performance and long-term stability. This review summarizes recent progress in strategies to address these challenges, focusing on controlling key issues such as Sn^2+^ oxidation, rapid crystallization, and high defect densities. Specific approaches discussed include precursor purification, the use of chemical additives, compositional engineering, surface modifications, interlayers, and the design of charge-transporting materials. Future advances will require the development of thicker high-quality absorber layers, robust electron- and hole-selective contacts, improved operational durability, and scalable fabrication methods. These integrated strategies will be essential for realizing efficient, stable, and large-area lead-free perovskite photovoltaics.

## Abstract 2

Coordinated control of oxidation, composition, crystallization, surface defects, and charge-transport layers is essential for advancing tin perovskite solar cells toward efficient, stable, and scalable devices.

## Graphical abstract

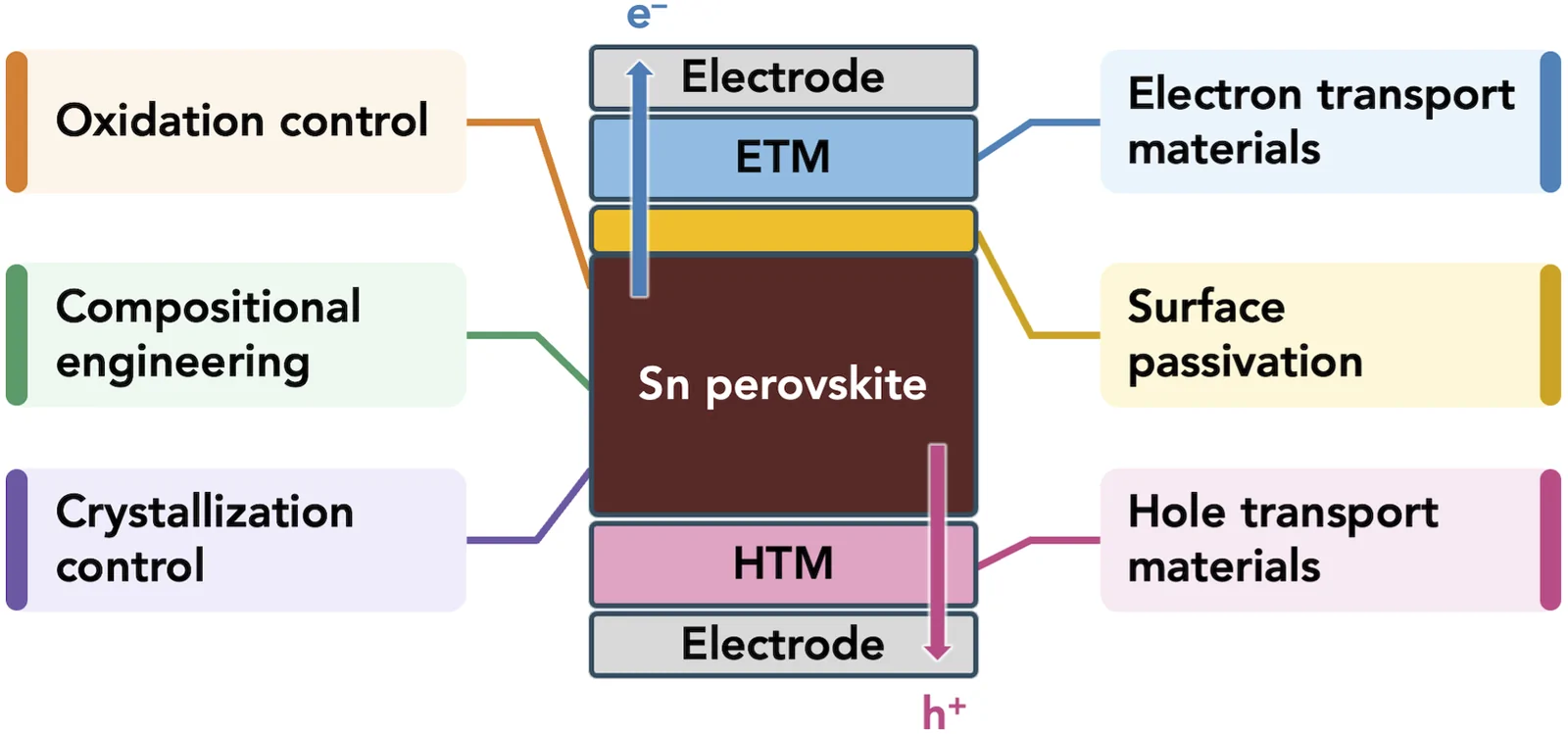

## 1. Introduction

Over the past decade, metal halide perovskites have attracted considerable research interest due to their high optical extinction coefficients, tunable band gaps, defect tolerance, low processing temperature, and solution processability.^1–3^ The power conversion efficiency (PCE) of perovskite solar cells (PSCs) has skyrocketed from 3.8% (ref. 4) to certified values now reaching 28%,^5–8^ comparable to those of currently commercially viable crystalline silicon solar cells.^9^

Despite these advantages and achievements, the success of high-performance metal halide PSCs relies on the use of lead (Pb) as their central element. In the event of cell damage from physical impact and the failure of cell encapsulation, Pb, which is neurotoxic, may leach into the environment in the form of water-soluble Pb compounds, presenting environmental and health concerns.^10^ Due to these concerns, researchers have focused on exploring alternatives to Pb halide perovskites, including those adopting ABX_3_ (A: monovalent cation; B: divalent cation; X: halide) perovskite structures and perovskite-derived structures (Fig. 1a). Halide double perovskites and vacancy-ordered perovskites have also emerged as promising stable Pb-free photovoltaic materials, although further advances in band-structure engineering, optical absorption, and carrier transport are needed to improve their photovoltaic performance.^11,12^ Among all candidates, tin (Sn) halide perovskites are considered one of the most promising alternatives,^13^ as Sn and Pb share similar outer electronic configurations and ionic radii. This enables divalent tin (Sn^2+^) to be incorporated in an ABX_3_ perovskite crystal structure and causes Sn halide perovskite to exhibit numerous properties similar to those of Pb halide perovskites. Although many insights can be translated from Pb perovskites, currently the PCE of the most efficient Sn halide perovskite solar cells (Sn PSCs) is ∼18%,^14–16^ lagging behind that of Pb-based devices. The long-term stability of Sn PSCs also remains less established than that of Pb-based devices. This gap stems from challenges that arise when replacing Pb with Sn, namely the inherent instability of the Sn^2+^ cation and its rapid crystallization kinetics that often results in poor film quality. To overcome these challenges, researchers have proposed various strategies to enhance the performance and stability of Sn PSCs. While previous reviews have comprehensively discussed the fundamental properties, defect chemistry, degradation mechanisms, and stabilization strategies of Sn halide perovskites,^17–19^ this review highlights recent progress in pure Sn-based PSCs from a materials-to-device design perspective. We focus on precursor purification, redox control, crystallization regulation, compositional engineering, surface modification, and electron- and hole-transporting materials, with an emphasis on how these approaches translate into improved device efficiency and stability.

**Fig. 1:**
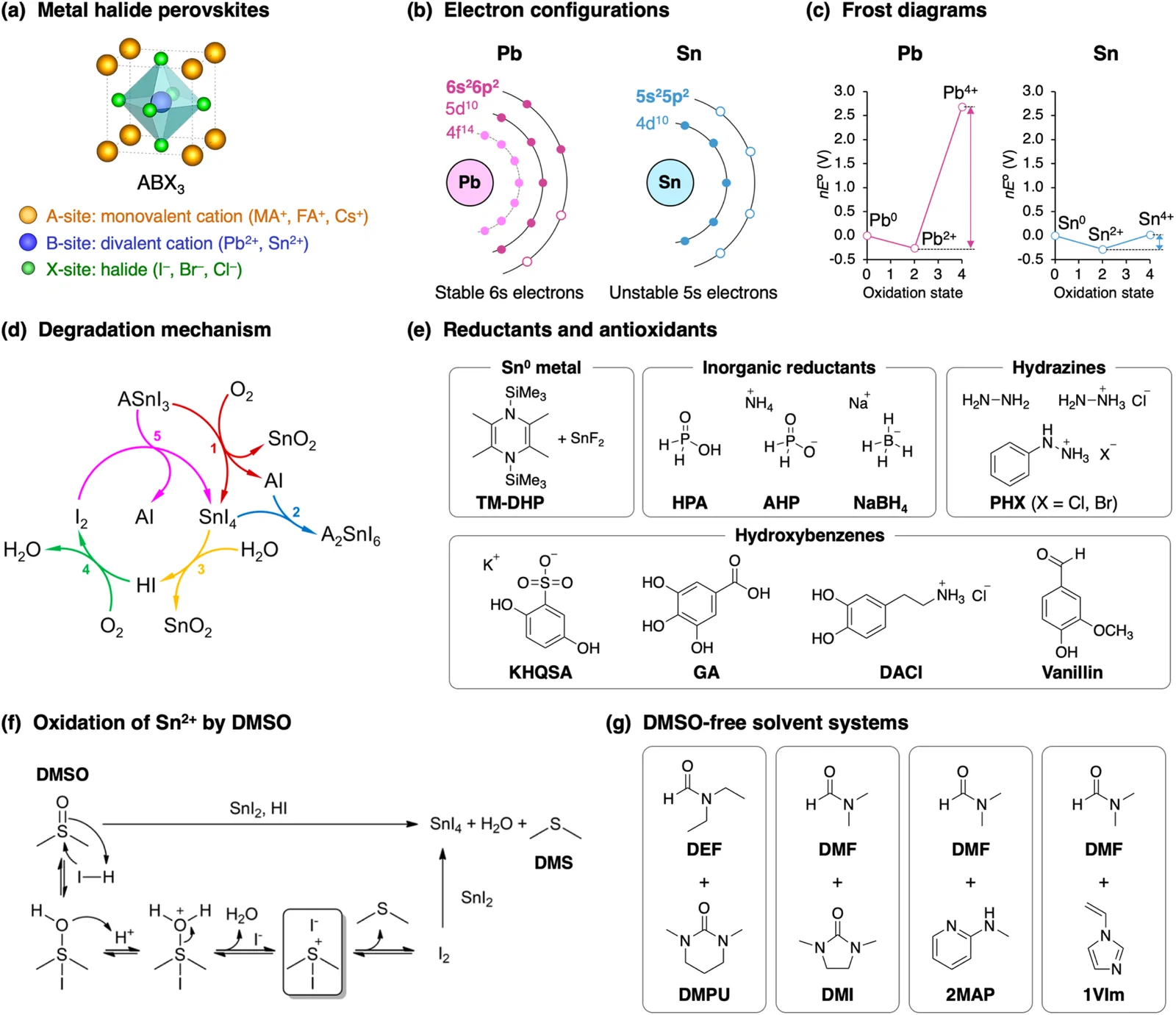
Oxidation issues in Sn-based perovskites. (a) Structure of metal halide perovskites. (b) Electron configurations for Pb and Sn atoms. (c) Frost diagrams for Pb and Sn. Data were taken from ref. 13. (d) Degradation mechanism of Sn perovskite under exposure to ambient air. Adapted with permission from ref. 26. Copyright 2021 the authors, licensed under the Creative Commons Attribution 4.0 International License (CC BY). (e) Chemical structures of reported reductants and antioxidants to suppress Sn^2+^ oxidation. (f) Oxidation mechanism of Sn^2+^ by dimethylsulfoxide (DMSO) solvent. Adapted with permission from ref. 58. Copyright 2020 the authors, licensed under the Creative Commons Attribution 3.0 Unported Licence (CC BY). (g) Reported DMSO-free solvent systems to fabricate Sn perovskite films.

## 2. Oxidation issue

### 2.1 Purification of starting materials

Pb and Sn are both group 14 elements, with Pb in the 6th period ([Xe]4f^14^5d^10^6s^2^6p^2^) and Sn in the 5th period ([Kr]4d^10^5s^2^5p^2^) (Fig. 1b). Upon forming divalent species, Pb^2+^ benefits from the lanthanide contraction: weak f-orbital shielding stabilizes the 6s orbital, lowering its energy. In contrast, Sn^2+^ lacks this effect, leaving its 5 s orbital relatively high in energy and more easily ionized.^13^ The relative stability of Pb and Sn in their various oxidation states compared to their elemental forms can be evaluated using Frost diagrams (Fig. 1c). Compared to Pb^2+^, Sn^2+^ occupies a much shallower thermodynamic sink, reflecting its lower stability and greater susceptibility to oxidation to higher valence states under external influences.^20–22^ Oxidation of Sn^2+^ to tetravalent tin (Sn^4+^) in Sn perovskite films induces p-type doping and the formation of electron trap sites, both of which degrade solar cell performance.^23,24^

The purity of Sn^2+^ in the starting materials directly influences the quality of the resulting Sn perovskite films. Wakamiya and co-workers reported that commercially available SnI_2_ reagents often contain a considerable amount of Sn^4+^ impurities.^25,119^ Sn nuclear magnetic resonance (NMR) and thermogravimetric analysis (TGA) revealed the presence of ∼10 wt% SnI_4_, even in SnI_2_ with a nominal purity of 99.9% on a trace-metal basis. Purification by sublimation under reduced pressure removed SnI_4_, yielding red crystalline SnI_2_, though traces of SnO_2_ remained. Further purification *via* recrystallization from *N*,*N*-dimethylformamide (DMF) or dimethyl sulfoxide (DMSO) affords solvent-coordinated complexes, [SnI_2_(dmf)] or [SnI_2_(dmso)], respectively. Similar solvent-coordinated Sn complexes with other halides such as [SnBr_2_(dmf)], [SnBr_2_(dmso)_2_], [SnCl_2_(dmf)], and [Sn_2_F_4_(dmso)_2_] can be synthesized in the same manner. These complexes, while air-sensitive, are stable under inert conditions and serve as pure, convenient precursors for Sn perovskites. Islam, Haque, and co-workers identified SnI_4_ as a key degradation intermediate in Sn perovskites.^26^ SnI_4_ rapidly evolves into I_2_*via* hydrolysis and oxidation, establishing a cyclic degradation process: I_2_ aggressively oxidizes the perovskite, forming more SnI_4_ and accelerating decomposition (Fig. 1d). They showed that removing SnI_4_ by sublimation improves film quality and stability. Fang, Ke and co-workers reported that SnI_4_ impurities can be removed simply by washing SnI_2_ powder with toluene, reducing Sn^4+^ content in perovskite films.^27^ FASnI_3_ (FA^+^ = formamidinium) films prepared from purified SnI_2_ exhibited improved morphology, enhanced crystallinity, longer carrier lifetimes, and lower trap-state densities. Devices using purified SnI_2_ showed a PCE of 10.8% (11.06% after aging), outperforming control devices (9.8%).

Methods to remove Sn^4+^ impurities from the perovskite precursor solutions have also been developed. Liu, Bian, and co-workers reported that adding Sn^0^ powder to the FASnI_3_ precursor solution reduces Sn^4+^ to Sn^2+^*via* the redox reaction: Sn^4+^ + Sn^0^ → 2Sn^2+^.^28^ Systematic optimization of stirring time and annealing conditions significantly improved film morphology, reduced trap-state density, and enhanced carrier lifetime. Devices fabricated with purified SnI_2_ achieved a PCE of 6.8%, comparable to those fabricated from SnI_2_ with a high purity of 99.999%. Later, Ning and co-workers developed a one-step synthesis (OSS) route to form SnI_2_·(DMSO)_*x*_ adducts directly from metallic Sn and I_2_.^29^ Sn^0^ powder can also remove trace-metal contaminants from perovskite precursor solutions. Meggiolaro, Snaith, and co-workers showed that Bi^3+^ can occur as a trace impurity in commercial precursors and introduce deep electron traps in mixed Pb–Sn perovskites, causing pronounced photoluminescence quenching at concentrations as low as 1 ppm. Sn^0^ reduces dissolved Bi^3+^ to insoluble Bi^0^ (3Sn^0^ + 2Bi^3+^ → 3Sn^2+^ + 2Bi^0^), which can be removed together with excess Sn^0^ by filtration.^30^ Thus, Sn^0^ treatment can purify precursor solutions by both converting Sn^4+^ into Sn^2+^ and scavenging harmful metal impurities. Although demonstrated for mixed Pb–Sn inks, this result underscores the importance of controlling trace-metal contamination in Sn-based precursor systems.

Wakamiya and co-workers developed a Sn^4+^ scavenging method using a strong reductant with an 8π electron system, 1,4-bis(trimethylsilyl)-2,3,5,6-tetramethyl-1,4-dihydropyrazine (TM-DHP, Fig. 1e).^31^ TM-DHP reacts selectively with tin fluoride (SnF_2_) to generate Sn^0^ nanoparticles, which have higher reactivity compared to bulk Sn^0^ powder due to their high surface area. These nanoparticles aggregate in the precursor solution and can be removed by filtration. X-ray photoelectron spectroscopy (XPS) confirmed that Sn^4+^ content in the bulk perovskite decreased to nearly zero. Optimal treatment with 1 mol% TM-DHP increased photoluminescence (PL) lifetime of FA_0.75_MA_0.25_SnI_3_ (MA^+^ = methylammonium) film from 4.0 ns to 14.3 ns and improved device PCE from 8.1% to 9.9%. Later, Yamaguchi, Marumoto and co-workers investigated band bending in perovskite solar cells using electron spin resonance (ESR) spectroscopy. The addition of Sn powder^32^ or TM-DHP^33^ was found to suppress Sn^4+^ formation in the Sn perovskite film, induce upward band bending at the interface between poly(3,4-ethylenedioxythiophene): polystyrene sulfonate (PEDOT:PSS) and Sn perovskites, and reduce interfacial recombination.

### 2.2 Reductants and antioxidants

In addition to purifying starting materials, various reductants and antioxidants have been explored as additives in perovskite precursor solutions to mitigate the Sn^2+^ oxidation issues.

The most widely used additive in Sn PSCs is SnF_2_, with optimal concentration typically below 20 mol%. Mhaisalkar, Mathews and co-workers demonstrated that CsSnI_3_ (Cs^+^ = cesium) delivers high photocurrents (>22 mA cm^−2^) when Sn vacancies are suppressed by SnF_2_ additive.^34^ This vacancy modulation enabled the first functional CsSnI_3_ devices with a PCE of 2.0%. Zhao, Xiong, Yan and co-workers further improved device performance by employing FASnI_3_ absorbers, which exhibit higher phase stability, combined with 10 mol% SnF_2_.^35^ They achieved a PCE of 6.2%. Seo, Seok and co-workers addressed phase separation caused by excess SnF_2_ by introducing pyrazine ligands to form a SnF_2_–pyrazine complex, ensuring homogeneous dispersion of SnF_2_ and reducing Sn^4+^ formation.^36^ Regarding the role of SnF_2_, theoretical studies by Meggiolaro, Angelis and co-workers revealed that the low ionization potential of MASnI_3_ stabilizes tin vacancies (V_Sn_^2−^) and interstitial iodine (I_i_^−^), causing severe self-p-doping and promoting Sn^2+^ → Sn^4+^ oxidation.^37^ They proposed that Sn-rich growth conditions by SnF_2_ addition can raise the ionization potential and suppress defect formation. Pascual, Abate and co-workers elucidated the chemical mechanism of SnF_2_, showing that SnF_2_ complexes Sn^4+^ as SnF_4_*via* ligand exchange, preventing the incorporation of SnI_4_ into the perovskite lattice and reducing oxidation-driven defects.^38^ Additionally, SnF_2_ has been reported to act as nucleation seeds that enhance the coverage and crystallinity of tin perovskite films,^39^ while reacting with PEDOT:PSS at the bottom interface to form an ultrathin SnS interlayer.^40^ As an alternative to SnF_2_, Liu and co-workers replaced SnF_2_ with tin trifluoromethanesulfonate (Sn(OTf)_2_). Having higher solubility in perovskite solution than SnF_2_, Sn(OTf)_2_ delayed the crystal nucleation process and resulted in the enhancement of crystal growth, thus increasing the device PCE.^41^

As reductive additives to the perovskite precursor solution, Wang and co-workers demonstrated that incorporating hypophosphorous acid (HPA, H_3_PO_2_, Fig. 1e) into CsSnIBr_2_ precursors effectively suppresses Sn^4+^ formation and reduces Sn vacancies, yielding improved crystallinity, lower trap density, and enhanced device performance (PCE = 3.2%).^42^ Yan and co-workers later showed that ammonium hypophosphite (AHP, NH_4_^+^H_2_PO_2_^−^) not only acts as a reducing agent but also regulates crystallization, producing compact, pinhole-free films with reduced trap density.^43^ Devices incorporating 5 mol% AHP achieved PCEs of 7.3% and maintained 50% of initial PCE after 500 h in air. Sánchez, Mora-Seró and co-workers employed a reducing agent, sodium borohydride (NaBH_4_).^44^ The devices exhibited outstanding operational stability, preserving 96% of initial performance (10.6%) after 1300 h of continuous operation under N_2_.

Hydrazine derivatives represent another class of reductants. Kanatzidis and co-workers introduced a hydrazine (H_2_N–NH_2_) vapor atmosphere during film formation for MASnI_3_, CsSnI_3_, and CsSnBr_3_, achieving significant reductions in Sn^4+^/Sn^2+^ ratios, prolonged carrier lifetimes, and PCEs of 3.9% (MASnI_3_) and 3.0% (CsSnBr_3_).^45^ Islam and co-workers used hydrazinium chloride (H_2_N–NH_3_^+^Cl^−^) as a co-additive with SnF_2_, leveraging its dual role as a reducing agent and morphology controller.^46^ This approach suppressed Sn^4+^ formation by ∼20%, improved film coverage and crystallinity, resulting in a PCE of 5.4% with extended device lifetime (65% retention after 1000 h of storage in N_2_) compared to pristine devices. Liu and co-workers reported that phenylhydrazinium halide salts (PHX, X = Cl, Br) act as self-repairing trap passivators, reducing Sn^4+^ and healing Sn-vacancy defects under illumination.^47,48^ This approach yielded a PCE of 13.4% (certified 12.4%) and robust illumination stability, maintaining 82% of initial PCE after 330 h under continuous operation.^48^

Catechol derivatives (dihydroxybenzenes) have also been employed as antioxidants due to their reversible redox conversion between dihydroxy and diketone forms. Yan and co-workers employed hydroxybenzene sulfonic acids (*e.g.*, KHQSA) with excess SnCl_2_ to form a SnCl_2_-additive complex layer that encapsulates grains and scavenges oxygen (Fig. 1e).^49^ This antioxidant grain passivation strategy significantly reduced Sn oxidation, yielding PCEs up to 6.8% and unprecedented air stability (80% PCE retention after 500 h without encapsulation). The same group later demonstrated that gallic acid (GA) can further increase the PCE to 9.0%.^50^ Other catechol derivatives, such as dopamine hydrochloride (DACl)^51^ and vanillin,^52^ have similarly been reported to enhance both efficiency and stability against light and air exposure.

Recently, Wang and co-workers introduced a regenerative redox cycling strategy using 4-mercaptobenzoic acid (4-MBA).^53^ The thiol group of 4-MBA can reduce Sn^4+^ to Sn^2+^, forming 4,4′-dithiobisbenzoic acid (DTBA). The disulfide bond in DTBA can then be cleaved under ultraviolet (UV) illumination to regenerate 4-MBA. This strategy enables the continuous reduction of Sn^4+^ and resulted in a device with a PCE of 15.2%. The device also exhibited enhanced stability, showing negligible degradation after 1100 hours of day–night cyclic operation and retaining 92% of its initial PCE after 5000 h of storage in N_2_.

### 2.3 Solvents

Until now, DMF and DMSO remain the most widely used solvents for preparing Sn halide perovskites.^54,55^ Kanatzidis and co-workers demonstrated that using DMSO as a coordinating solvent forms a SnI_2_·3DMSO intermediate, slowing crystallization and enabling uniform, pinhole-free MASnI_3_ films.^56^ While DMSO facilitates film formation by coordinating with precursors, its oxidative nature has emerged as a critical stability concern. Kanatzidis, Sargent and co-workers revealed that DMSO can oxidize Sn^2+^ to Sn^4+^ at temperatures above 100 °C.^57^ Using ^1^H NMR and X-ray absorption near-edge spectroscopy (XANES), they confirmed that this redox reaction produces dimethyl sulfide (DMS) and Sn^4+^, even under inert conditions. Dallmann, Abate and co-workers employed solid- and solution-state ^119^Sn NMR to determine when and how Sn^2+^ oxidation occurs during processing.^58^ Their study revealed that Sn^4+^ is absent in freshly prepared solutions but forms during annealing in DMSO at elevated temperatures under acidic conditions. The oxidation proceeds *via* a redox reaction between DMSO and Sn^2+^, likely involving an iododimethylsulfonium iodide intermediate, with protons acting as catalysts (Fig. 1f).

These findings underscore the necessity of developing DMSO-free solvent systems (Fig. 1g). Abate and co-workers introduced a one-step deposition of FASnI_3_ using an *N*,*N*-diethylformamide (DEF)/*N*,*N*′-dimethylpropylene urea (DMPU) solvent system,^59^ achieving PSCs with 8.7% PCE.^60^ Nazeeruddin, Dyson and co-workers used the same solvent system to achieve efficient perovskite light-emitting devices.^61^ Adldanmasy, Musiienko, Abate and co-workers addressed rapid crystallization in DMSO-free DMF/1,3-dimethyl-2-imidazolidinone (DMI) solvent systems by adding 4-*tert*-butylpyridine (*t*BP) and piperazine dihydriodide (PDAI).^62^ These additives form pre-nucleation clusters that slow crystal growth and improve film quality, resulting in a 7.8% PCE and high shelf stability (>3000 h). Recently, Kitamura, Hayase and co-workers replaced DMSO with 2-methylaminopyridine (2MAP), which strongly coordinates with SnI_2_ (Δ*H* = −3.35 eV), suppressing Sn^4+^ formation and enabling solar cells with a 9.7% PCE.^63^ When combined with a tin oxide (SnO_*x*_) layer prepared using atomic layer deposition (ALD), the device showed superior stability at 85 °C. Nakamura, Wakamiya and co-workers further advanced this field by developing a substrate-independent, antisolvent- and DMSO-free fabrication route referred to as the vacuum-quenching with crystal growth regulator (V-CGR) method.^64^ This technique employs 1-vinylimidazole (1VIm) to form amorphous [SnI_2_-(1VIm)] intermediates, enabling controlled crystallization and pinhole-free films on diverse substrates. The V-CGR method yielded PCEs up to 12.1% and demonstrated large-area scalability (7.5 × 7.5 cm^2^ films and 21.6 cm^2^ modules) with improved thermal stability (81% retention after 100 h at 85 °C).

## 3. Compositional engineering

### 3.1 A-site compositions

MA^+^, FA^+^, and Cs^+^ are the most commonly used A-site cations in Sn-based perovskites. The choice of A-site cation influences the band gap of Sn perovskite.^65^ For instance, substituting FA^+^ with smaller Cs^+^ in FA_1−*x*_Cs_*x*_SnI_3_ contracts the lattice, increases Sn–I orbital overlap, and reduces the band gap (Fig. 2a).^66^ Defect formation is also strongly affected by the A-site cation. Comparing FASnI_3_ with MASnI_3_, the larger ionic radius of FA^+^ weakens the antibonding interaction between I 5p and Sn 5 s orbitals, resulting in higher Sn vacancy formation energies and tunable conductivity (from p-type to intrinsic) in FASnI_3_. In contrast, MASnI_3_ exhibits a low Sn vacancy formation energy, leading to persistent p-type behavior.^67^ Liu, Bian and co-workers demonstrated that composition engineering through mixed organic cations (FA^+^/MA^+^) enhances film morphology, suppresses recombination, and improves PCE to 8.1% for FA_0.75_MA_0.25_SnI_3_.^68^ Wu and co-workers reported that Cs^+^ incorporation into FASnI_3_ reduces the tolerance factor toward unity and contracts the lattice, thereby improving crystallinity and reducing trap density. Devices with 8% Cs^+^ doping achieved a PCE of 6.1%.^69^ Nishimura, Hayase and co-workers revealed a strong correlation between lattice strain, carrier mobility, and device performance.^70^ Reducing strain *via* A-site cation substitution from Na_0.1_(FA_0.75_MA_0.25_)_0.9_SnI_3_ to EA_0.1_(FA_0.75_MA_0.25_)_0.9_SnI_3_ (EA^+^ = ethylammonium) improved PCE from 1.5% to 5.4%. Very recently, Ning and co-workers extended A-site compositional engineering to high-entropy wide-bandgap (1.67 eV) Sn perovskites by incorporating five organic A-site cations (thiophene-2-ethylammonium, formamidinium, dimethylammonium, ethylammonium, guanidinium), which enhanced the stability of perovskite under humid conditions and thus enabled the integration of atomic layer deposition of SnO_*x*_ as an electron transport layer. The strategy enabled an 8.45% single-junction device as well as the first four-terminal Sn-perovskite/silicon tandem solar cell with a PCE of 16.2%.^71^

**Fig. 2:**
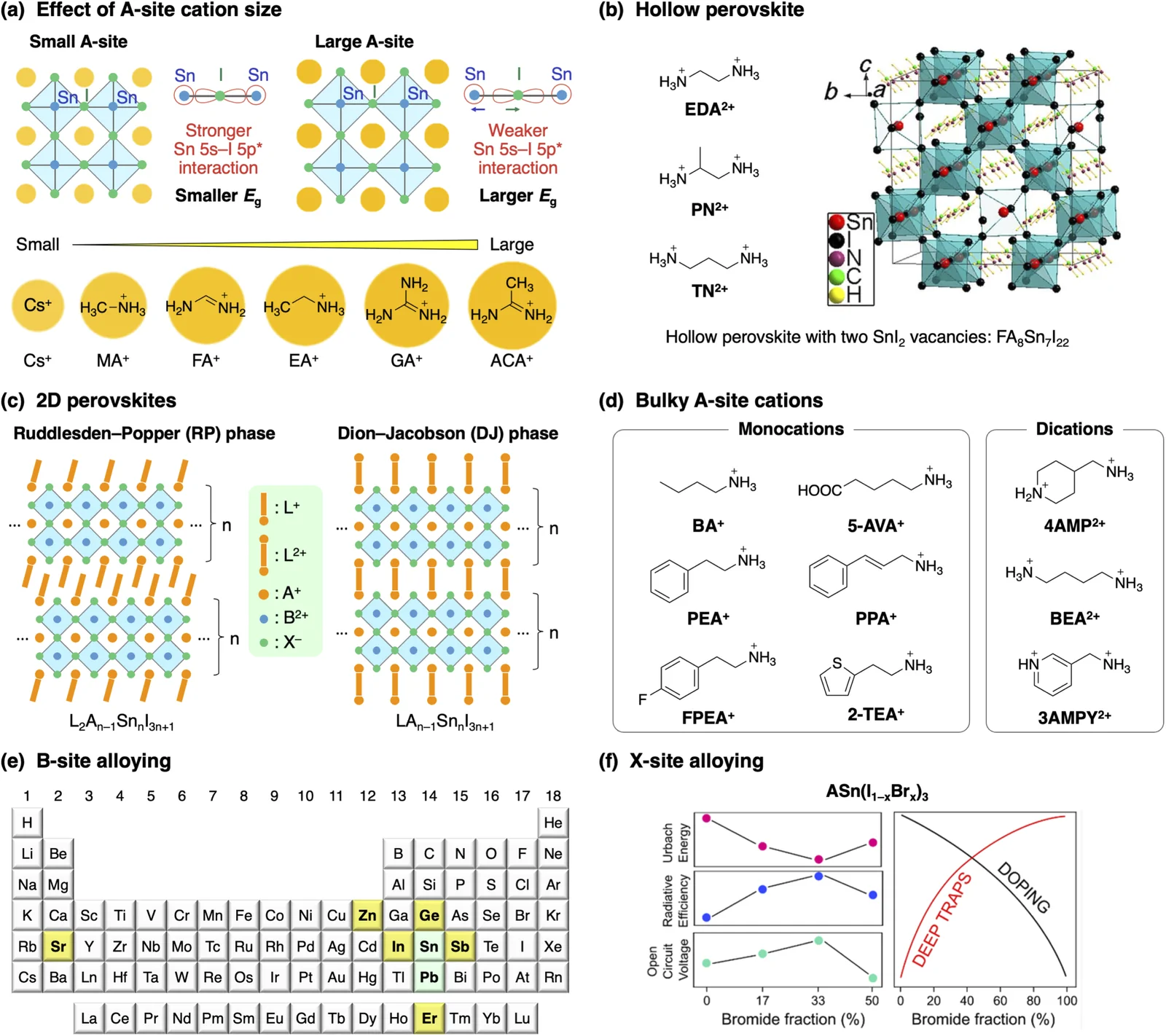
Composition engineering for Sn-based perovskites. (a) Chemical structures of A-site cations. (b) Structure of hollow perovskite with SnI_2_ vacancies. Adapted with permission from ref. 73. Copyright 2017 the authors, licensed under the Creative Commons Attribution 4.0 International License (CC BY-NC). (c) Schematic structures of two-dimensional (2D) perovskites. (d) Chemical structures of reported bulky cations to construct 2D perovskites. (e) Periodic table showing elements alloyed with Sn in yellow. (f) Effect of bromide (Br) mixing to iodide-based Sn perovskites. Adapted with permission from ref. 118. Copyright 2023 the authors, licensed under the Creative Commons Attribution 4.0 International License (CC BY).

Beyond these conventional cations, Kanatzidis and co-workers demonstrated that incorporating ethylenediammonium (EDA^2+^) into FASnI_3_ forms a hollow structure with SnI_2_ vacancies, enabling improved film coverage and suppressed Sn^2+^ oxidation (Fig. 2b).^72^ Devices achieved a PCE of 7.1% and a *V*_OC_ of 0.48 V. The same group applied EDA^2+^ to MASnI_3_, achieving 6.6% PCE with significantly enhanced air stability compared to pristine MASnI_3_.^73^ They later introduced propylenediammonium (PN^2+^) and trimethylenediammonium (TN^2+^) to FASnI_3_. Devices incorporating 10% PN^2+^ or TN^2+^ achieved PCEs of 5.9% and 5.5%, respectively, compared with 2.5% for pristine FASnI_3_.^74^ Diau and co-workers demonstrated that incorporating EDAI_2_ into FASnI_3_ significantly improved film quality and stability by passivating surface defects and suppressing Sn^2+^/Sn^4+^ oxidation.^75^ Notably, EDAI_2_ induced a slow passivation effect, enabling PCE to increase from ∼6% to 8.9% after prolonged storage. The same group introduced guanidinium (GA^+^) into FASnI_3_ with 1 mol% EDAI_2_, achieving optimized lattice stability, reduced defect states, and favourable band alignment with transport materials.^76^ This hybrid cation strategy delivered certified PCEs of 8.3% (fresh) and 9.6% after 2000 h. Later, the group achieved a PCE of 14.5% and high shelf stability (>90% retention after 3000 h) by co-incorporating acetamidinium (ACA^+^, 10 mol%) and rubidium (3 mol%).^77^ Hayase and co-workers employed partial substitution of FA^+^ with EA^+^ to tune the tolerance factor toward unity, reducing lattice strain and aligning energy levels with transport materials. This approach suppressed trap density by an order of magnitude and boosted PCE to 13.2% with a *V*_OC_ of 0.84 V.^78^ Similarly, Loi and co-workers used EAI as an additive, rather than a substitute for FAI, to improve the quality of tin perovskite films.^79^ Saeki and co-workers employed a machine-learning-assisted multivariate analysis using stable A_2_SnI_6_ pseudoperovskites to predict the performance of ASnI_3_ systems incorporating mixed ternary and quaternary A-site cations, providing a data-driven approach to compositional optimization.^80^

### 3.2 Quasi-2D and 2D/3D structures

When large organic cations (L^+^ or L^2+^) that cannot fit into the A-site of a three-dimensional (3D) perovskite lattice are introduced, low-dimensional perovskites (2D, 1D, or 0D) are formed (Fig. 2c). Two major classes of 2D perovskites are the Ruddlesden–Popper (RP) phase, L_2_A_*n*−1_Sn_*n*_I_3*n*+1_, and the Dion–Jacobson (DJ) phase, LA_*n*−1_Sn_*n*_I_3*n*+1_, where *n* represents the number of [SnI_6_]^4−^ octahedral layers separated by spacer cations (L^+^).^81^

Kanatzidis and co-workers first demonstrated layered RP 2D Sn-based perovskites using butylammonium (BA^+^), which enhances moisture resistance and suppresses p-type self-doping (Fig. 2d).^82^ By controlling film orientation through solvent engineering, compact morphologies and improved durability were achieved, with BA_2_MA_3_Sn_4_I_13_ (*n* = 4) solar cells reaching a PCE of 2.5% and higher stability compared to 3D MASnI_3_. Zhang, Ning and co-workers demonstrated that incorporating phenylethylammonium (PEA^+^) into FASnI_3_ forms low-dimensional perovskites with highly oriented films, enabling perpendicular growth that facilitates charge transport and suppresses oxidation.^83^ Devices based on PEA_2_FA_8_Sn_9_I_28_ (*n* = 9) exhibited a PCE of 5.9%. Loi and co-workers further optimized the ratio of PEA^+^ and FA^+^, where PEA_2_FA_24_Sn_25_I_76_ (*n* = 25) yielded the highest PCE of 9.0%.^84^ Ning and co-workers reported that by using ammonium thiocyanate (NH_4_SCN) as a pseudohalide additive to regulate crystallization of PEA_0.15_FA_0.85_SnI_3_, 2D-quasi-2D-3D hierarchical structure can be formed on ITO/NiO_*x*_ substrate where parallel-oriented 2D PEA_2_SnI_4_ layers overlay 3D FASnI_3_.^85^ This architecture improved oxidation resistance, reduced carrier density, and enhanced mobility, achieving a PCE of 9.4%.

The use of PEA-based 2D/3D perovskites has become a central strategy in Sn PSC research after Ning and co-workers reported that combining PEA_0.15_FA_0.85_SnI_3_ perovskite with indene-C_60_ bisadduct (ICBA) electron-transporting material (ETM) yielded a high *V*_OC_ of 0.94 V.^86^ Ning, Mi and co-workers later reported that incorporating Lewis base additive, trimethylthiourea (3T), can regulate nucleation, enhance grain overlap, and passivate Sn^2+^/I^−^ defects.^87^ He and co-workers introduced 4-fluorophenethylammonium bromide (FPEABr) to form 2D/3D heterogeneous perovskite microstructures, delivering a PCE of 14.8%.^88^ Similarly, Mi and co-workers reported that the FPEABr causes an interfacial dipole between Sn perovskites and ICBA, improving energy alignment at the interface.^89^ This modification raised *V*_OC_ from 0.88 V to 0.97 V and achieved a PCE of 15.7%. Recently, Chen, Wang and co-workers introduced Cs^+^ ions which partially replaced bulky PEA^+^ in colloidal electrical double layers, reducing aggregation barriers and promoting homogeneous 2D/3D microstructures with improved crystallinity and orientation.^15^ This strategy delivered a PCE of 17.1% (certified 16.7%) with *V*_OC_ = 0.99 V and high stability, retaining over 92% of initial PCE after 1500 h of continuous illumination. A similar strategy was adopted by Yang, Bao and co-workers.^90^ In contrast to Chen, Wang and co-workers, who used CsI as an additive in the PEA_0.15_FA_0.85_SnI_2.85_Br_0.15_ perovskite solution, Yang, Bao and co-workers used CsI to partially replace FAI in their PEA_0.15_FA_0.85_SnI_3_ perovskite. The device with CsI substitution exhibited a PCE of 13.5% and enhanced stability, showing a negligible decrease in PCE after 5800 h of storage under N_2_. Most recently, Xu, Chen, Wang, and co-workers partially substituted Cs^+^ with PEA^+^ in CsSnI_3_-based perovskites, inducing the formation of a PEA_2_CsSn_2_I_7_ 2D template. Owing to its close lattice match with 3D γ-CsSnI_3_, this template promoted the growth of the γ phase and suppressed the formation of non-perovskite δ-CsSnI_3_.^91^ This approach enabled PEA_0.4_Cs_0.6_SnI_2.6_Br_0.4_-based PSCs with a PCE of 15.3%, which retained over 95% of their initial PCE after 1280 h of operation.

Beyond conventional BA^+^ and PEA^+^ spacers, Yuan and co-workers reported quasi-2D perovskites using 5-ammoniumvaleric acid (5-AVA^+^) as the organic spacer.^92^ Assisted by NH_4_Cl additives to induce vertical crystal orientation, (5-AVA)_2_FA_4_Sn_5_I_16_ (*n* = 5) devices showed a PCE of 8.7%. Kanatzidis, Wu and co-workers synthesized a conjugated organic cation, 3-phenyl-2-propen-1-ammonium (PPA^+^), to mitigate the insulating nature of BA^+^ and PEA^+^.^93^ The Sn PSC using PPA_0.15_FA_0.85_SnI_3_ exhibited a higher PCE of 9.6% compared to the PEA-based device. Ripolles, Abargues, Boix and co-workers introduced thiophene-2-ethylammonium halides (2-TEAX) as crystallization modulators and defect passivators in FASnI_3_.^94^ Devices incorporating 2-TEABr reached 12.0% PCE with improved voltage and sustained >95% efficiency after 2000 h under continuous illumination. Wang, Zhang, Yan and co-workers recently compared the isomers of thiophene-based spacers (2-TEA^+^ and 3-TEA^+^).^95^ Compared to 2-TEA^+^ and PEA^+^, 3-TEA^+^ produced denser crystal packing of [SnI_6_]^4−^ octahedral layers, achieving a higher PCE of 14.2%.

In addition to RP phases, DJ-phase 2D Sn perovskites have gained attention due to their stronger interlayer bonding and reduced Sn vacancy formation. Zhou, Padture and co-workers pioneered DJ-phase 2D Sn perovskites, using 4-(aminomethyl)piperidinium (4AMP^2+^).^96^ Devices using (4AMP)(FA)_3_Sn_4_I_13_ (*n* = 4) delivered 4.2% PCE with improved operational stability. Zhang, Song and co-workers developed DJ-phase perovskites using 1,4-butanediammonium (BEA^2+^) ligands, achieving high crystal symmetry, weakened quantum confinement, and efficient carrier transport.^97^ Solar cells based on (BEA)FA_2_Sn_3_I_10_ (*n* = 3) reached 6.4% PCE. Recently, Liu, Yang, Liang and co-workers introduced 3-(aminomethyl)pyridinium (3AMPY^2+^)-based DJ perovskites, where a small amount of (3AMP)SnI_4_ added to PEA-containing 2D–3D systems modulated crystallization and phase distribution.^98^ This approach yielded high-quality films with low trap density and optimized band alignment, improving the PCE from 10.9% to 13.3%.

### 3.3 B-site compositions

Analogous to the B-site alloying employed in mixed Sn–Pb perovskites,^99–101^ partial substitution of Sn^2+^ with other metal cations has also been explored in Sn-based perovskites.

Being in the same group as Sn and Pb on the periodic table while exhibiting low toxicity, germanium (Ge^2+^) has been a popular option (Fig. 2e). Hayase and co-workers first demonstrated Ge^2+^ incorporation into Sn PSCs.^102^ With the optimal concentration of germanium iodide (GeI_2_) as additive, the device exhibited enhanced PCE and shelf stability in air. Since then, GeI_2_ has been adopted by several different research groups as additive to improve the performance of Sn PSCs.^27,103,104^ Ge^2+^ mixing was also shown to be effective in suppressing the formation of Sn^4+^ in inorganic tin halide perovskites (CsSn_1−*x*_Ge_*x*_I_3_) prepared by vacuum deposition.^105,106^ We note that the precise mechanism by which Ge^2+^ improves the efficiency of Sn PSC remains unclear and requires further study. For example, Ju and co-workers revealed that in their PEA_0.15_FA_0.85_SnI_3_, Ge^2+^ is more concentrated near the top surface of Sn perovskite.^107^ In contrast, Fu and co-workers found that most of their Ge^2+^ in their EDA_0.01_FA_0.98_SnI_3_ perovskite accumulated near or merged with the underlying PEDOT:PSS layer.^108^

Regarding alloying Sn with other metal elements, Musiienko, Abate and co-workers doped their EDA_0.01_FA_0.78_MA_0.20_SnI_3_ with strontium (Sr^2+^) by adding strontium iodide (SrI_2_) to their perovskite precursor solution. The similar ionic radii of Sr^2+^ and Sn^2+^ allow Sr^2+^ to fill the Sn^2+^ vacancies, thus reducing the self p-doping and the microstrain in the crystal structure.^109^ The optimal concentration of SrI_2_ increased the device PCE from 6.3% to 7.5%. A similar strategy to reduce microstrain in the crystal structures was adopted by Kwak, Kim and co-workers, who substituted Sn^2+^ with zinc ions (Zn^2+^) by adding Zn powder into the perovskite precursor solution.^110^ Due to the redox potential difference between zinc and tin, Zn^2+^ formed and partially substituted for Sn^2+^ in the crystal structure. The device PCE was thus improved from 8.6% to 11.4%.

In addition to isovalent doping, aliovalent cations can also be used to improve the performance of Sn PSCs. For instance, Song and co-workers incorporated erbium chloride (ErCl_3_) into perovskite precursor solution, where ErCl_3_-DMSO may compete with SnI_2_-DMSO, thus increasing colloidal size in the perovskite precursor solution and regulating the nucleation/crystallization dynamics.^111^ The device containing the optimal ErCl_3_ concentration (1 mg mL^−1^) showed a PCE of 14.0% accompanied by enhanced stability, with the device retaining 80% of its initial PCE after 1300 h of photoaging. In a similar fashion, Wu and co-workers,^112^ and Yan and co-workers^113^ added indium bromide (InBr_3_, 0.1 mol%) and antimony trifluoride (SbF_3_, 0.01 mol%) to their respective perovskite precursor solutions, achieving PCEs of 14.0% and 14.37% and enhanced shelf stability, with devices retaining 96% and 95% of their initial PCE after 1500 h of storage.

### 3.4 X-site compositions

Incorporating bromide at the X-site effectively suppresses p-doping in iodide-based Sn perovskites. This modification also enables the formation of wide-bandgap Sn perovskites, making them suitable for tandem solar cells and indoor photovoltaic applications.

Mulmudi, Mathews and co-workers demonstrated that halide substitution in CsSnI_3_ (CsSnI_3−*x*_Br_*x*_) effectively tunes the optoelectronic properties, widening the band gap from 1.27 to 1.75 eV, reducing carrier density by nearly three orders of magnitude, and increasing the *V*_OC_ from 0.20 V to 0.41 V.^114^ Similarly, Seok, Seo, and co-workers reported that partial Br^−^ substitution in FASnI_3_ reduces carrier density by three orders of magnitude (6.8 × 10^17^ to 7.8 × 10^14^ cm^−3^), mitigates Sn-vacancy defects, and improves energy alignment with titanium oxide (TiO_2_), enabling a PCE of 5.5%.^115^ He and co-workers demonstrated that mixed-cation alloying (FA^+^/MA^+^) with Br^−^ incorporation in FASnI_3_ promotes highly oriented film growth along the (001) facet, lowers trap density, and reduces Sn^4+^ content by ∼40%, enabling a PCE of 9.3%.^116^ Wakamiya and co-workers systematically explored the compositional space of A-site cations (FA^+^, MA^+^, Cs^+^) and Br^−^/I^−^ ratios using high-purity SnBr_2_ precursors and Sn^4+^ scavenging strategies.^117^ They identified FA_0.75_MA_0.25_SnI_2.25_Br_0.75_ as the optimal composition, providing balanced energy-level alignment and prolonged carrier lifetime, leading to a PCE of 7.7%. This work underscores the importance of purity and controlled Br incorporation, as excessive bromide (>1 : 1 Br/I) shortens PL lifetimes and degrades performance. Petrozza and co-workers also demonstrated that moderate Br incorporation in MASn(Br_*x*_I_1−*x*_)_3_ tunes the band gap (1.3–2.2 eV) and suppresses self-doping by destabilizing Sn vacancies.^118^ Optimal Br fractions (∼33%) minimize the Urbach energy (∼33 meV) and maximize PL efficiency, correlating with improved *V*_OC_ in devices (Fig. 2f). Extending this concept to all-inorganic systems, Im, Hong and co-workers combined theoretical calculations with experiments to identify CsSnI_2_Br as an optimal composition, achieving a PCE of 11.9%.^119^ Recently, Vivo, Abate and co-workers fabricated FASnI_2_Br-based solar cells employing a DMSO-free DEF/DMPU solvent system, enabling an indoor PCE of 11.9% under 1000 lx white light-emitting diode illumination.^120^

Apart from halogen tuning, pseudohalides, especially thiocyanates (SCN^−^), have emerged as an appealing candidate for X-site modification due to their unique coordination chemistry and exceptional capability to modulate crystallization kinetics. Hao and co-workers found that for their 2D/3D Sn perovskites, strong coordination between SCN^−^ and Sn^2+^ can regulate the crystal growth process, accelerating the formation of high-*n*-value 2D phase perovskites.^121^ The coordination between SCN^−^ and Sn^2+^ also retarded the oxidation of Sn^2+^ and enhanced the stability of the perovskites. Similar strategies were also utilized by Zhou, Ning and co-workers, who partially replaced PEABr with PEASCN, enhancing the solar cell PCE from 13.4% to 14.6%.^122^

### 3.5 Additives to regulate crystal growth

Several additives have been reported to regulate the crystal growth of Sn-based perovskites. Ye, Priya and co-workers used phthalimide (PTM) as an additive in the CsSnI_3_ perovskite precursor solution.^123^ The lone electron pairs of NH and CO groups could coordinate with Sn^2+^, protecting Sn^2+^ from oxidation to Sn^4+^. With PTM, the device with active areas of 0.08 cm^2^ and 1 cm^2^ achieved PCEs of 10.1% and 7.8%, respectively. Liang and co-workers used 2-(2-aminoethyl)-2-thiopseudourea dihydrobromide (AET), which contains two amino (–NH_2_) groups, an imino (=NH) group, and a thioether (–S–) bond, to form coordination and hydrogen bonds with Sn perovskite precursors.^124^ This approach enabled solar cells with a PCE of 13.6% and a high fill factor (FF) of 0.80. Cho, Kang and co-workers employed 4-phenylthiosemicarbazide (4PTSC) as an additive to form a strong chemical coordination between SnI_2_ and the S=C–N, –NH_2_, and phenyl groups of 4PTSC.^125^ They found that compared with thiosemicarbazide (TSC), the phenyl group of 4PTSC helps increase the solubility and establish preferred crystal growth orientation. The optimal concentration of 4PTSC (1 mol% relative to Sn perovskite) enhanced the PCE of the PEA_0.15_FA_0.85_SnI_2_Br solar cell from 8.7% to 12.2%. Hu, Yang, Meng and co-workers utilized 3-aminopyrrolidine dihydroiodate (APDI_2_) as an additive in their PEA_0.15_FA_0.85_SnI_2.85_Br_0.15_ perovskites.^126^ The strong chemisorption of APD^2+^ into the Stern layer of perovskite colloids lowered the absolute zeta potential of the colloids in the perovskite precursor solution, while the colloidal size remained unchanged. This, in turn, reduced the interaction energy between colloidal particles and lowered the critical nucleation concentration, thereby accelerating nucleation and leading to improved film morphology. With the addition of APDI_2_, the PCE of the PEA_0.15_FA_0.85_SnI_2.85_Br_0.15_ device increased from 13.10% to 15.13%.

## 4. Surface modification

### 4.1 A- and X-site passivation

Compared to the bulk, the surfaces of Sn perovskite films typically contain a higher concentration of Sn^4+^ impurities and structural defects related to A-, B-, and X-site vacancies (Fig. 3a).^23,127,128^ Surface post-treatment, implemented *via* spin coating or vacuum deposition, has emerged as an effective strategy to mitigate these issues.

**Fig. 3:**
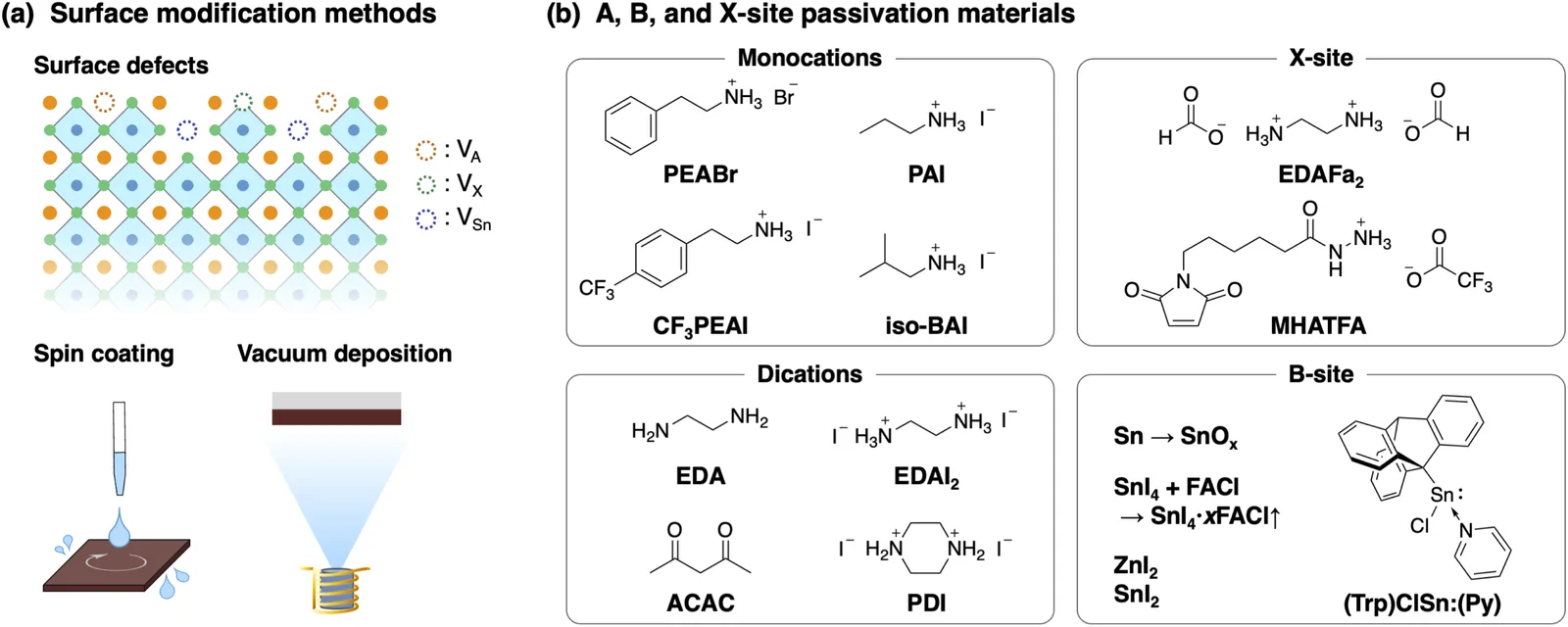
Surface modification of Sn-based perovskites. (a) Schematic illustration of surface defects and passivation methods using wet and dry process. (b) Chemical structures of materials used for surface post-treatments.

In addition to directly incorporating bulky ammonium cations into the precursor solution, surface-localized 2D/quasi-2D layers can be formed by sequential deposition. Diau and co-workers used hexafluoro-2-propanol as a second-step solvent to deposit various bulky ammonium iodides onto a preformed 3D Sn perovskite film without damaging the underlying layer.^129^ Its strong interaction with the bulky ammonium cations retarded surface conversion, enabling controlled formation of a thin 2D/quasi-2D capping layer. This layer protected the 3D grains from moisture infiltration and suppressed surface recombination. Among the investigated cations, anilinium afforded a PCE of 10.6% together with improved ambient, light-soaking, and thermal stability. Using a related surface-conversion strategy, Yao, He and co-workers demonstrated that interface engineering using phenylethylammonium bromide (PEABr) forms an ultrathin low-dimensional perovskite layer at the perovskite/ETM interface, effectively suppressing Sn^2+^ oxidation, improving crystallinity, and optimizing band alignment (Fig. 3b).^130^ Hao and co-workers introduced a post-deposition treatment using 3-(trifluoromethyl)phenethylammonium iodide (CF_3_PEAI).^131^ This treatment effectively suppresses interfacial trap states, relaxes residual tensile strain, and enhances hydrophobicity, boosting *V*_OC_ from 0.61 V to 0.73 V and PCE from 8.5% to 10.4%. Wang, Han and co-workers applied *n*-propylammonium iodide (PAI) to the perovskite surface before thermal annealing to induce the templated growth of FASnI_3_, thus improving the quality of the perovskite films.^132^ Similarly, Qin, Lu and co-workers reported that iso-butylammonium iodide (iso-BAI) treatment induces controlled surface recrystallization rather than 2D phase formation, significantly improving crystallinity, reducing microstrain, and suppressing oxidation.^133^ This strategy enhanced *V*_OC_ from 0.68 V to 0.72 V and PCE from 11.8% to 14.2%.

Besides monoammonium halides, diamine derivatives have also been employed for surface modification. Kamarudin, Hayase and co-workers demonstrated that post-treatment with ethylenediamine (EDA) effectively passivates undercoordinated Sn and removes residual SnI_2_, improving film morphology and reducing trap density.^134^ This strategy increased *V*_OC_ by ∼0.1 V and boosted PCE from 8.1% to 10.2%. Zhang, Hayase and co-workers introduced a sequential passivation approach using acetylacetone (ACAC) followed by EDA.^135^ This synergistic treatment suppressed trap-assisted recombination, improved carrier mobility, and delivered a 13.0% PCE with improved shelf stability (80% retention after 70 days). Snaith, Wakamiya and co-workers later revealed, using ^1^H NMR spectroscopy, that diamines employed for surface treatment react with FA^+^ in the perovskite to form diammonium species, rather than coordinating with metal ions.^136^ A recent study by Luther and co-workers suggests that the enhanced performance and stability following EDA treatment may originate from the conversion of the 3D perovskite surface into lower-dimensional phases.^137^ Wakamiya and co-workers developed a universal surface treatment using EDAI_2_, which forms a surface dipole and enhances energy alignment.^138–140^ Applied *via* either wet or dry processes, EDAI_2_ consistently improved *V*_OC_ (by up to 0.2 V) and efficiency across various compositions—raising Sn-based PSCs from 6.7% to 11.4% and Pb-based devices to 22%—while significantly enhancing shelf stability (>800 h without degradation). Recently, Wang, Wang and co-workers introduced a synergistic interfacial strategy combining piperazine dihydroiodide (PDI) and ferrocene (Fc) at the perovskite/C_60_ interface, where PDI forms interfacial dipoles to optimize band alignment and passivate defects, while Fc establishes π–π conjugation with C_60_, enhancing electron transport and suppressing Sn^2+^ oxidation.^141^ This dual modification boosted PCE from 10.6% to 13.7% and improved operational stability (>82% retention after 300 h illumination).

Regarding the X-site passivation, Ran, Xing, Xia and co-workers employed ethylenediamine formate (EDAFa_2_) as a bifunctional ionic salt for *in situ* passivation, which stabilized Sn^2+^ through strong coordination and reduced interfacial trap density by nearly half.^142^ Devices incorporating this layer achieved a PCE of 9.4% and retained ∼95% of their initial performance after 1960 h of storage in N_2_. Yin and co-workers developed a surface reconstruction method employing 6-maleimidohexanehydrazide trifluoroacetate (MHATFA), which passivates both cationic and anionic defects, reduces Sn^4+^ formation, and converts the perovskite surface from p-type to weakly n-type, creating a back-surface field that facilitates electron extraction.^143^ This dual chemical and electronic modulation raised *V*_OC_ to 0.80 V and PCE to 13.6%, with devices retaining over 76% of initial efficiency after 1000 h of light illumination.

### 4.2 B-site passivation

To address B-site-related defects, Wang, Hayase and co-workers proposed a bi-interface strategy by inserting Sn^0^ layers at both the bottom and top of the perovskite absorber, with the top layer converted into SnO_*x*_.^144^ This dual modification suppressed Sn^4+^ formation, reduced trap densities, and improved energy alignment, enabling a PCE of 14.3% with *V*_OC_ = 0.77 V. Wang, Zhou and co-workers deposited a thin layer (3 nm) of FACl on the as-spun Sn perovskites *via* vacuum evaporation. The FACl reacted with Sn^4+^ on the perovskite surface, forming a volatile SnI_4_·*x*FACl complex, which was subsequently removed during post thermal annealing.^145^ The device with FACl de-doping the perovskite surface exhibited a PCE of 14.7%, with the device retaining 92% of its initial PCE after 1000 h of storage. Chen, Wakamiya and co-workers employed various subnanometer vacuum-deposited metal halide interlayers (ZnI_2_, SnI_2_, SnBr_2_, GeI_2_, *etc.*) between FA_0.75_MA_0.25_SnI_3_ and C_60_, narrowing the electron accumulation width in C_60_ and reducing interfacial recombination without chemical passivation.^146^ This approach—specifically, the use of a ZnI_2_ layer—boosted *V*_OC_ by up to 0.2 V and improved PCE from 10.2% to 12.8% for FA_0.75_MA_0.25_SnI_3_, with similar gains for FASnI_3_ and 2D/3D mixed Sn perovskites, while enhancing shelf stability. Similarly, Hayase and co-workers formed a SnI_2_ passivation layer on the surface of Sn perovskites to suppress the non-radiative recombination between Sn perovskites and the ETM by removing some of the MAI in the perovskite bulk during thermal annealing at 100 °C.^63^ Nakamura, Wakamiya and co-workers recently developed solution-processable chlorostannylene complexes, particularly the pyridine adduct (Trp)ClSn:(Py), as Sn^2+^ sources for surface passivation. Applied using nonpolar solvents, this treatment effectively reduced p-doping and suppressed nonradiative recombination at the perovskite/C_60_ interface, increasing *V*_OC_ from 0.56 V to 0.65 V and PCE from 9.3% to 10.5%.^147^

## 5. Electron-transporting materials (ETMs)

### 5.1 Conventional fullerene derivatives: C_60_, PCBM, and ICBA

The 5 s and 5p orbital energies in Sn are higher than the 6s and 6p orbital energies in Pb, resulting in higher-lying valence band maximum (*E*_VB_) and conduction band minimum (*E*_CB_) levels in Sn perovskites compared to their Pb counterparts (Fig. 4a).^148^ The resulting upward-shifted band edges not only affect defect formation and self-p-doping in Sn perovskites,^37^ but also lead to energy-level mismatches with conventional charge-transport materials. For instance, the lowest unoccupied molecular orbital (LUMO) level of C_60_ (−4.21 eV)^31^ is significantly deeper than the *E*_CB_ of FASnI_3_ (−3.60 eV),^117^ leading to substantial *V*_OC_ losses when C_60_ is used as an ETM (Fig. 4b). Therefore, ETMs with shallower LUMO levels are desirable. An effective approach to tune the LUMO level of fullerene derivatives is to functionalize the C=C bonds of the C_60_ cage, as the cleavage of a C=C bond raises the LUMO level by approximately 0.2 eV.^149,150^ Phenyl-C_61_-butyric acid methyl ester (PCBM) and ICBA are typical mono- and bis-functionalized fullerene derivatives used to mitigate *V*_OC_ loss in Sn PSCs.

**Fig. 4:**
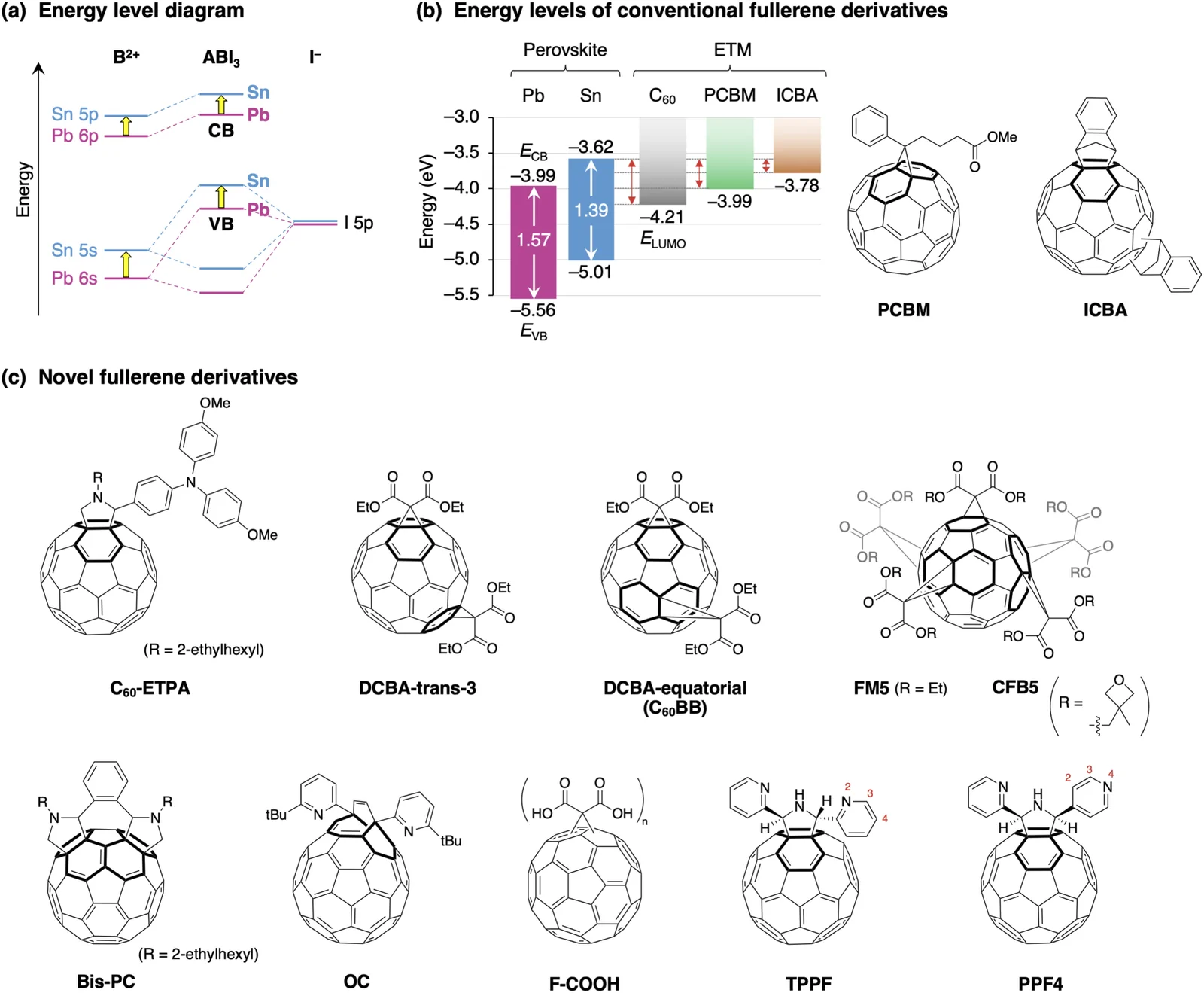
Electron-transporting materials (ETMs) for Sn PSCs. (a) Schematic energy levels comparing the APbI_3_ and ASnI_3_ perovskites. Adapted with permission from ref. 148. Copyright 2019 the authors, licensed under the Creative Commons Attribution 4.0 International License (CC BY). (b) Energy level diagram comparing the conduction band minimum (*E*_CB_) of Pb and Sn-based perovskites and LUMO energy (*E*_LUMO_) of conventional fullerene derivatives. (c) Chemical structures of reported novel fullerene derivatives for Sn PSCs.

Ning and co-workers addressed the severe *V*_OC_ deficit by introducing ICBA as an ETM, whose shallower LUMO level improved energy alignment, reducing interfacial recombination.^86^ This strategy enabled an unprecedented *V*_OC_ of 0.94 V and a certified PCE of 12.4%. Similarly, Chung and co-workers reported that the *V*_OC_ trend (C_60_ < PCBM < ICBA) mirrors the order of LUMO levels, confirming the dominant role of energy offsets in voltage loss.^151^ Wakamiya and co-workers demonstrated that reducing energy disorder in the ICBA layer *via* solvent engineering and optimized annealing significantly suppresses non-radiative recombination at the perovskite/ETM interface.^152^ This approach yielded *V*_OC_ values up to 1.01 V—only 0.15 V below the Shockley–Queisser limit for *E*_g_ = 1.44 eV. Mi and co-workers further addressed morphological inhomogeneity of ICBA by blending 1% PMMA with ICBA.^153^ Combined with D-sorbitol that reconfigured the PEDOT:PSS backbone and boosted its hole conductivity, these modifications led to a PCE of 15.8%.

Although C_60_ has a deeper LUMO than PCBM or ICBA, it offers higher electron mobility and suitability for thermal evaporation. Higher electron mobility ensures rapid electron sweep out in the Sn PSCs, while the thermal evaporation allows C_60_ to be conformally deposited at a lesser thickness than those of PCBM and ICBA ETMs. This, in turn, reduces parasitic light absorption by the ETMs in the Sn PSCs. To suppress the non-radiative recombination between Sn perovskite and C_60_, Hayase and co-workers inserted a thin interlayer consisting of a mixture of ICBA, PCBM, and poly(3-hexylthiophene) (P3HT) between Sn perovskites and C_60_ ETMs.^154^ ICBA served as an electron transport channel, PCBM enhanced the electronic properties, and P3HT controlled the morphology of the interlayer, respectively. The device with PCBM:ICBA:P3HT interlayer exhibited a PCE of 14.6%, which was further enhanced to 15.3% when 5-mercapto-1-methyltetrazole tin salt (SnT_2_) was used as an additive for the perovskite precursor solution.^104^

### 5.2 Novel fullerene derivatives

Despite the advantages of ICBA, it consists of up to 18 possible regioisomers depending on the position of the second addition (*e.g.*, *cis*-1 to 3, *trans*-1 to 4, equatorial).^155,156^ Using a mixture of these isomers introduces energetic disorder, whereas isolating a specific isomer requires a costly purification process.^157^ Therefore, the development of well-defined fullerene derivatives with high-lying LUMO levels is highly desirable.

Wu, Hao and co-workers demonstrated that raising the LUMO level of fullerene derivatives by introducing an electron-donating triarylamine moiety can alleviate voltage losses in Sn PSCs, with C_60_-ETPA-based devices achieving a *V*_OC_ of 0.76 V and a PCE of 10.2%.^158^ Tian, Meng, Wei and co-workers reported that the number of isomers can be reduced to six in diethylmalonate-C_60_ bisadduct (DCBA) (Fig. 4c).^159^ They systematically investigated four well-defined regioisomers of DCBA, revealing that molecular structure strongly influences energy levels, packing, and interfacial interactions. Among them, the *trans*-3 isomer exhibited the most favourable combination of shallow conduction band minimum (−3.71 eV), amorphous morphology, and high electron mobility (1.03 × 10^−4^ cm^2^ V^−1^ s^−1^), enabling a certified PCE of 14.3%. Later, the same group developed a steric-hindrance-assisted synthesis to obtain equatorial isomers of C_60_ and C_70_ bisadducts (C_60_BB, C_70_BB) in near-quantitative yield, combined with solvent engineering to control molecular packing.^160^ Films processed with anisole formed dense amorphous layers with superior electron mobility, delivering PCEs of 14.5% (C_60_BB) and 14.3% (C_70_BB). They further synthesized multidentate fullerene molecules with 3 to 6 diethylmalonate groups (FM3–FM6) and employed them as interfacial layers between Sn perovskite and PCBM.^161^ The pentasubstituted derivative (FM5) exhibited optimal coordination with Sn^2+^ and a favourable LUMO level, enabling efficient charge extraction and moisture resistance. Devices using FM5 achieved 15.05% PCE and retained >90% efficiency after 300 h of storage in air and >95% after 5000 h of storage in N_2_. Nakamura, Wakamiya and co-workers introduced a phenylene-bridged bis(pyrrolidino)fullerene (Bis-PC), a single isomer derivative that eliminates the complexity of mixed-isomer bisadducts like ICBA.^162^ This molecular design raised the LUMO level and reduced voltage loss. Combined with perovskite compositional engineering, Bis-PC afforded a *V*_OC_ of 0.86 V and a PCE of 12.3%, while maintaining shelf stability for over 3000 hours. Murata, Wakamiya and co-workers also reported an open-cage bis[60]fulleroid (OC), offering a shallower LUMO level (−3.98 eV) than PCBM and superior thermal stability (up to 450 °C) than ICBA.^163^ OC-based devices achieved 9.6% efficiency in solution-processed Sn PSCs and 7.6% when OC was deposited using vacuum deposition. More recently, Wei, Tian and co-workers developed *in situ* cross-linked multi-anchoring fullerene interlayers that form robust solvent-resistant networks at the perovskite/ETM interface, passivating interfacial defects and improving both efficiency and operational stability.^164^ The optimized CFB5-modified devices reached a PCE of 15.7% and retained 96% of their initial efficiency after 800 h under continuous illumination. Going beyond fullerene-based materials, Li, Liang and co-workers reported fullerene-free Sn PSCs using fluorinated triple-acceptor polymer ETMs.^165^ This strategy enabled PCEs of 16.1% for small-area (0.04 cm^2^) devices and 14.7% for 1 cm^2^ devices, with certified values of 15.9% and 14.5%, respectively, highlighting the potential of polymer ETMs for scalable Sn-based photovoltaics.

Fullerene derivatives have also been applied as additives to control Sn perovskite crystallization. Khadka, Takeoka, Sahara, Shirai and co-workers demonstrated that fullerene-based multifunctional molecules (F–COOH, F–OH, F–OSO_3_H) effectively stabilize Sn^2+^ and passivate defects, with F–COOH delivering the highest performance improvement (PCE from 8.2% to 11.2%) and enhanced operational stability by moderating crystallization and reducing trap states.^166^ Tian, Wei and co-workers developed a *trans*-isomeric pyridyl-substituted pyrrolidinofullerene (TPPF) additive that coordinates with multiple Sn colloids, slowing crystallization, passivating defects, and improving energy-level alignment.^167^ Using PCBM as an ETM, this approach delivered a PCE of 15.4% (certified 15.1%) with *V*_OC_ = 0.86 V. They subsequently synthesized *cis*-isomers with 2-, 3-, and 4-pyridyl groups (PPF2–PPF4) as precursor additives to regulate crystallization kinetics, with PPF4 inducing ordered colloid stacking and reducing defect density, while a C_60_ bis-adduct (C_60_BB) interlayer further optimized energy-level alignment.^14^ This strategy delivered a 16.05% PCE (certified 15.9%) and exceptional stability (99% retention after 600 h of operation).

### 5.3 Inorganic ETMs in n-i-p devices

Although the number of studies on regular (n-i-p) Sn PSCs is significantly lower than those on inverted (p-i-n) architectures, several ETMs have been developed for this configuration.

An early work by Kanatzidis and co-workers employed a zinc sulfide (ZnS) coating on TiO_2_ to create a stepped conduction band structure that facilitated electron extraction and suppressed interfacial recombination.^168^ This strategy increased the *V*_OC_ from 0.29 V to 0.38 V and improved the PCE to 5.3%. Yokoyama and co-workers demonstrated that *V*_OC_ loss in Sn PSCs is strongly governed by conduction band offset at the ETM/perovskite interface.^169^ By employing Nb_2_O_5_ bearing a higher *E*_CB_ than TiO_2_, they achieved a *V*_OC_ of 0.42 V and a PCE of 5.1%. Recently, Liang and co-workers addressed the inherent limitations of conventional metal oxide ETMs, where oxygen vacancies promote Sn^2+^ oxidation.^170^ They introduced metal chalcogenide ETMs, particularly Sn(S_0.92_Se_0.08_)_2_, which combine shallow *E*_CB_, high conductivity, and high chemical stability. This approach boosted *V*_OC_ to 0.73 V and PCE to 11.78%, while delivering exceptional operational stability (>95% retention after 1632 h).

The development of n-i-p Sn PSCs is hindered by chemical and electronic incompatibility between metal-oxide ETMs and Sn precursors. During two-step deposition, mesoporous TiO_2_ can promote Sn^2+^ oxidation, while direct SnO_2_/SnI_2_ contact can induce interfacial redox reactions; mixed-valence SnO_*x*_ may also exhibit ambipolar transport and hole leakage. Diau and co-workers addressed these issues by combining Cl-doped SnO_2_ with a multifunctional polybenzoxazine treatment to control the surface oxidation state, passivate defects, suppress interfacial redox reactions, and improve electron selectivity.^171^ This enabled a sequentially deposited planar n-i-p device with a PCE of 3.81%, demonstrating the importance of controlling the oxidation state, ambipolarity, and chemical reactivity of metal-oxide ETMs.

## 6. Hole-transporting materials (HTMs)

### 6.1 Bulk HTMs

Due to its high conductivity and suitable energy level for Sn perovskites, PEDOT:PSS is currently the most frequently used hole-transporting material (HTM) in Sn PSCs (Fig. 5a).^172^ However, the acidic nature of PEDOT:PSS may accelerate the oxidation of iodide to iodine in perovskites, thus limiting the stability of Sn PSCs. To overcome this limitation, various alternative HTMs have been proposed.

**Fig. 5:**
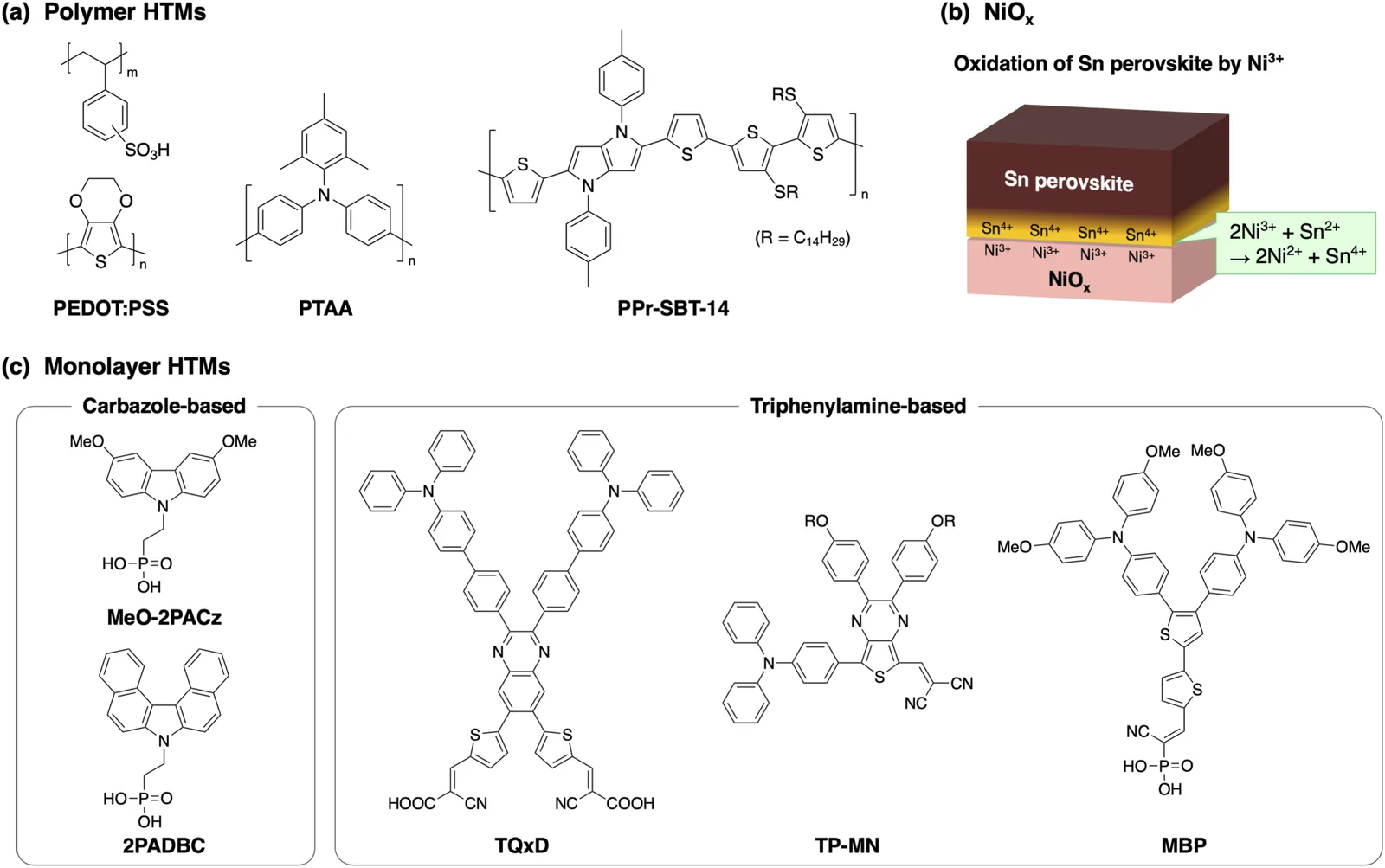
Hole-transporting materials (HTMs) for Sn PSCs. (a) Chemical structures of polymer HTMs. (b) Oxidation of Sn perovskite by Ni^3+^ at the perovskite/NiO_*x*_ interface. (c) Chemical structures of reported monolayer HTMs.

Abate and co-workers explored water-free PEDOT-complex as an alternative for the commonly used aqueous PEDOT:PSS. To address the low wettability of water-free PEDOT for the perovskite solution, they decorated the surface of PEDOT-complex with Al_2_O_3_ nanoparticles, achieving DMSO-free solar cells with a PCE of 7.5%.^173^ Recently, they further improved the device performance to 11% by inserting a thin layer of PEAI between Al_2_O_3_ nanoparticles and the perovskite layer.^174^ The device exhibited a decent storage stability, retaining ∼90% of its initial PCE after 3500 h of storage in N_2_.

Bis(4-phenyl)(2,4,6-trimethylphenylamine) (PTAA) has been widely used as an HTM in Pb perovskites.^175^ However, the hydrophobicity of the PTAA surface and the rapid crystallization rate of Sn perovskites make it difficult to grow high-quality Sn perovskite on PTAA. Diau and co-workers successfully addressed this issue by modifying the PTAA surface with PEAI to make it more hydrophilic.^176^ This surface modification, combined with a two-step growth method where SnI_2_ and FAI were sequentially deposited, improved the device PCE from 1.5% to 8.3%.

As dopant-free organic HTMs, Chen, Diau and co-workers developed PPr-SBT-14, a pyrrolopyrrole (PPr)-based polymer containing thioalkylated bithiophene units.^177^ This material provided excellent energy-level alignment, hydrophobicity, and film morphology. Devices incorporating PPr-SBT-14 achieved 7.6% PCE and exhibited unprecedented shelf stability over 6000 h without encapsulation in a glovebox, far surpassing conventional PEDOT:PSS-based systems. More recently, Cheng, Diau, and co-workers developed benzodipyrrole-based heptacyclic ladder polymers as dopant-free HTMs for inverted Sn PSCs.^178^ PBDP-Ph exhibited favourable energy-level alignment, high hole mobility, and good interfacial compatibility, while optimization of its solution-state aggregation improved HTM smoothness and subsequent perovskite crystallization. Combined with two-step perovskite deposition, PBDP-Ph afforded a PCE of 9.1%, outperforming the PEDOT:PSS-based control device, and retained approximately 80% of its initial PCE after 5000 h of ISOS-D-1 storage and 90% of its peak output during a 5 h maximum-power-point test.

Nickel oxide (NiO_*x*_) has also been widely investigated as an alternative HTM. However, the surface of NiO_*x*_ typically contains Ni^3+^ species that can oxidize Sn perovskite at the interface (Fig. 5b). Incorporating quasi-2D Sn perovskites has been shown to mitigate this interfacial oxidation issue. Wang, Abate and co-workers used NiO_*x*_ HTM and introduced PEACl to FASnI_3_ precursors, enabling the formation of vertically oriented 2D RP phases that suppress Sn^2+^ oxidation and improve film morphology, achieving a PCE of 9.1%.^179^ Padture and co-workers advanced this concept by incorporating a bulky divalent cation, 4AMP^2+^ (4-(aminomethyl)piperidinium), to functionalize grain surfaces and boundaries, reducing defect-mediated recombination and oxygen ingress.^180^ This strategy yielded a PCE of 10.9%. Yan and co-workers engineered a vertically stratified 2D/3D heterojunction *via* vacuum-assisted spin coating, combined with guanidinium thiocyanate (GASCN) additive to enhance 2D-phase crystallinity.^181^ This architecture achieved a *V*_OC_ of 1.01 V with a minimal voltage loss of 0.39 V and a PCE of 13.8%. Chen, Padture and co-workers reported another strategy of doping FASnI_3_ with Ge^2+^, which induced an *in situ* GeO_2_ native oxide at the NiO_*x*_ interface, preventing direct contact between Sn perovskites and Ni^3+^.^182^ This passivating layer stabilized Sn^2+^, improved adhesion, and delivered a 10.4% PCE for flexible devices, with exceptional bending durability, maintaining 80% of its initial PCE after 2500 bending cycles.

Tin oxide (SnO_*x*_) represents another class of inorganic HTM for Sn PSCs. Hayase and co-workers demonstrated that a p-type SnO_*x*_ layer can be prepared either by Sn^0^ deposition followed by plasma treatment^183^ or from commercially available SnO_2_ nanoparticles,^184^ enabling hole collection and improving stability. Fukata, Islam and co-workers advanced this concept by synthesizing mixed-valence SnO_*x*_ nanoparticles *via* a synproportionation reaction.^185^ By carefully controlling the stoichiometry, the energy levels and the functionality of SnO_*x*_ as n-type or p-type can be adjusted. The device using p-type SnO_*x*_ nanoparticles as the HTM showed a device PCE comparable to that of the device using PEDOT:PSS, together with significantly enhanced thermal and operational stability.

### 6.2 Hole-collecting monolayers

Monolayer-based HTMs, such as [2-(9*H*-carbazol-9-yl)ethyl]phosphonic acid (2PACz) and phosphonic acid-functionalized triazatruxene (PATAT), have driven recent progress in inverted (p-i-n) Pb-based PSCs due to their high optical transparency and low electrical resistance.^186–190^ However, their application in Sn-based PSCs remains limited because their hydrophobic nature hinders the uniform formation of Sn perovskite films.^191^

Diau and co-workers pioneered this approach by employing a two-step sequential deposition method.^192^ It enabled the formation of a uniform Sn perovskite film on MeO-2PACz, achieving a PCE of 6.5% (Fig. 5c). The same group designed X-shaped quinoxaline-based HTMs with dual anchoring groups (CN/COOH) and thiophene π-extensions. Their best-performing molecule, TQxD, delivered an 8.3% PCE.^193^ They also reported that a simple benzoic acid derivative, 4-aminobenzoic acid, can work as a hole-selective monolayer, yielding 7.6% PCE.^194^ Yu and co-workers addressed the intrinsic *V*_OC_ deficit of monolayer-based Sn PSCs by using a mixture of MeO-2PACz and 6-phosphonohexanoic acid (6PA) that balances hydrophobicity and wettability, enabling uniform perovskite lamination and vertical crystal orientation.^195^ Coupled with an optimized antisolvent-assisted one-step deposition and surface passivation with ICBA, this holistic approach delivered a *V*_OC_ of 0.83 V and PCE of 9.4%. Xiao, Wang, Meng and co-workers introduced phytic acid dipotassium (PADP), a monolayer material with multiple phosphonic acid groups that strongly interacts with tin perovskite, enabling rapid nucleation and controlled crystal growth.^196^ This tailored interface produced uniform films and achieved a PCE of 12.5%, retaining 90% efficiency after 2000 h of continuous illumination.

Monolayer materials have also been explored as interfacial modifiers for NiO_*x*_. Zhu and co-workers introduced a dibenzocarbazole-based monolayer material, 2PADBC, which anchors Ni^3+^ sites and donates electrons to the perovskite, increasing the activation energy for Sn oxidation and reducing nonradiative recombination.^197^ This strategy boosted *V*_OC_ from 0.712 V to 0.825 V and improved PCE from 11.39% to 14.19%, while ensuring >93% efficiency retention after 1000 h under continuous illumination. Chen, Diau and co-workers developed three thienopyrazine-based monolayer materials (TP-MN, TP-CA, TPT-MN) with CN/CN or CN/COOH anchoring groups for NiO_*x*_ modification.^198^ Among them, TP-MN delivered the best performance (7.7% PCE) and shelf stability (∼80% retention after 4000 h), attributed to improved crystallinity and reduced recombination. They also developed cost-effective I-shaped monolayer materials featuring selenophene linkers and phosphonic anchoring groups.^199^ Most recently, a new efficiency record for Sn PSCs was established by Xu, Liang, Qi, and co-workers.^16^ By introducing MBP, an HTM containing dimethoxytriphenylamine groups and phosphonic acid anchoring groups, onto NiO_*x*_, they formed a homogeneous buried interface with optimized energy-level alignment and superior wetting properties. This strategy enabled record PCEs of 17.9% (certified 17.7%), while maintaining >94% operational stability after 1550 h. A comprehensive discussion of hole-collecting monolayers in Sn perovskite solar cells has been summarized in a recent review by Song, Diau and co-workers.^200^

### 6.3 HTM-free configurations

An alternative approach to improving the stability of the hole-collecting interface is to eliminate the HTM altogether. Lin, Diau, and co-workers demonstrated that a thin 2D Sn perovskite layer capping the 3D absorber creates a vertical 2D/3D p–n heterojunction with favourable energetic alignment.^201^ This internal heterojunction facilitated selective hole extraction without a separate HTM, yielding a PCE of 5.17% and stable operation for more than 200 h. Further advances in HTM-free Sn PSCs have focused on engineering the direct ITO/perovskite contact. Han and co-workers induced upward band bending using heterogeneous ammonium salts, achieving a PCE exceeding 10% with 95% retention after 40 days of light soaking and 90% after 300 h at 80 °C.^202^ Diau and co-workers modified ITO with nanoparticles and thermal/UV-ozone treatments, yielding a PCE of 9.7% and over 90% retention after 10 000 h of storage.^203^ More recently, Wu and co-workers used chlorine-radical treatment to passivate oxygen vacancies and tune the ITO work function, reducing the interfacial barrier by 0.20 eV and achieving a PCE of 14.2% with enhanced stability over 2000 h.^204^ These studies highlight buried-interface engineering as the key to efficient and stable HTM-free Sn PSCs.

## 7. Conclusions and outlook

Over the past decade, Sn-based PSCs have progressed rapidly from low-efficiency Pb-free alternatives to a distinct class of photovoltaic devices with steadily improving performance. Representative device parameters of selected Sn halide perovskite solar cells discussed in this review are summarized in Table 1. This progress has been enabled by the combined development of precursor purification, reducing agents and antioxidants, crystallization control, compositional engineering, surface passivation, and charge-transport-layer design. Most recently, the PCE of Sn-based PSCs reached 17.9%, with a certified value of 17.7%, through homogeneous buried-interface engineering in inverted devices.^16^ This result represents an important milestone, demonstrating that both efficiency and stability can be substantially improved through rational interfacial design.

**Table 1:** Performance summary of representative Sn halide perovskite solar cells

Key strategy	Structure	Additive	*J*_SC_ (mA cm^−2^)	*V*_OC_ (V)	FF (%)	PCE (%)	Ref.
Toluene-purified SnI_2_	ITO/PEDOT:PSS/FASnI_3_/C_60_/BCP/Ag	EDABr_2_, GeI_2_, SnF_2_	23.46	0.60	78.1	11.06	27
One-step synthesized SnI_2_·(DMSO)_*x*_	ITO/PEDOT:PSS/PEA_0.15_FA_0.85_Sn I_2.85_Br_0.15_/ICBA/BCP/Ag	SnF_2_, NH_4_SCN	20.6	0.91	77.1	14.63	29
*In situ* formed Sn^0^ nanoparticles to remove Sn(iv) impurities	ITO/PEDOT:PSS/FA_0.75_MA_0.25_SnI_3_/EDA/PCBM/C_60_/BCP/Ag	SnF_2_, TM-DHP	22.0	0.76	69	11.5	31
4-MBA additive as a regenerative reductant	FTO/PEDOT:PSS/FASnI_3_/C_60_/BCP/Ag	4-MBA, FPEABr, SnF_2_	22.51	0.95	70.9	15.15	53
1VIm additive regulating crystallization growth kinetics	ITO/MeO-2PACz/Al_2_O_3_ NPs/EDA_0.01_FA_0.98_SnI_3_/ICBA:PCBM/C_60_/BCP/Ag	1VIm, SnF_2_, NH_4_SCN	24.2	0.68	74	12.1	64
DMSO-free solvent system & Al_2_O_3_ nanoparticles enhancing wettability of water-free PEDOT-complex for perovskite solution	ITO/PEDOT-complex/Al_2_O_3_ NPs/FASnI_3_/C_60_/BCP/Ag	EDAI_2_, SnI_2_	20.2	0.535	69	7.5	173
DMSO-free solvent system & PEAI interlayer on Al_2_O_3_ nanoparticles	ITO/PEDOT-complex/Al_2_O_3_ NPs/PEAI/FASnI_3_/C_60_/BCP/Ag	2-Pyridyl thiourea, EDAI_2_, SnI_2_	21.3	0.69	75	11.0	174
A-site engineering	ITO/PEDOT:PSS/EDA_0.01_AC_0.1_ Rb_0.03_FA_0.86_SnI_3_/C_60_/BCP/Ag	Sulfamic acid, EDAI_2_, SnF_2_	23.9	0.84	72	14.5	77
3T additive regulating nucleation and growth kinetics	ITO/PEDOT:PSS/PEA_0.15_FA_0.85_ SnI_3_/ICBA/BCP/Ag	3T, *N*-methylthiourea	20.3	0.92	77	14.0	87
FPEABr additive inducing surface dipole	ITO/PEDOT:PSS/TEA_0.15_FA_0.85_ SnI_3_/ICBA/BCP/Ag	FPEABr, 3T, *N*-methylthiourea	21.7	0.974	74.1	15.7	89
CsI additive regulating nucleation and growth kinetics of Sn perovskite	ITO/PEDOT:PSS/PEA_0.15_FA_0.85_ SnI_2.85_Br_0.15_/ICBA/BCP/Ag	CsI, SnF_2_, NH_4_SCN	23.07	0.99	74.8	17.13	15
A-site engineering	ITO/PEDOT:PSS/PEA_0.4_Cs_0.6_ SnI_2.6_Br_0.4_/ICBA/BCP/Ag	MAAc, SnF_2_, NH_4_SCN	23.68	0.90	71.6	15.27	91
Gradiently distributed GeI_2_-induced vertical gradient homojunction	ITO/PEDOT:PSS/PEA_0.15_FA_0.85_ SnI_2.85_Br_0.15_/ICBA/BCP/Ag	GeI_2_, SnF_2_	20.9	0.85	75	13.3	107
GeI_2_ additive serving as nucleation sites and a protective layer	ITO/PEDOT:PSS/EDA_0.01_FA_0.98_ SnI_3_/C_60_/BCP/Ag	GeI_2_, SnF_2_	21.25	0.826	77.1	13.48	108
Sb^3+^ counter-doping oxidized tin perovskite	ITO/NiO_*x*_/PEA_0.2_FA_0.9_SnI_3_/ICBA/BCP/Ag	SbF_3_, SnF_2_	20.92	0.938	73.2	14.37	113
Thiocyanate pseudohalide regulating nucleation and growth kinetics of perovskite	ITO/PEDOT:PSS/FA_0.75_MA_0.25_ SnI_2.75_Br_0.25_/C_60_-ETPA/BCP/Ag	PEASCN, SnF_2_	21.17	0.863	70.5	12.88	121
Thiocyanate pseudohalide regulating nucleation and growth kinetics of perovskite	ITO/PEDOT:PSS/PEA_0.175_FA_0.825_ SnI_2.825_Br_0.15_SCN_0.025_/ICBA/BCP/Ag	SnF_2_, NH_4_SCN	20.6	0.905	78.6	14.6	122
Additive (APDI_2_) regulating the interaction between colloids in perovskite solution and thus accelerating nucleation	ITO/PEDOT:PSS/PEA_0.15_FA_0.85_ SnI_2.85_Br_0.15_/NCBA/BCP/Ag	APDI_2_, SnF_2_	21.58	0.97	72.3	15.13	126
Vacuum-deposited metal halide interlayer between Sn perovskite and ETM	ITO/PEDOT:PSS/FA_0.75_MA_0.25_ SnI_3_/ZnI_2_/C_60_/BCP/Ag	SnF_2_, NH_4_SCN	22.9	0.72	78	12.8	146
ETM with a higher LUMO level than conventional C_60_ and PCBM	ITO/PEDOT:PSS/PEA_0.15_FA_0.85_ SnI_3_/ICBA/BCP/Ag	SnF_2_, NH_4_SCN	17.4	0.94	75	12.4	86
PCBM:ICBA:P3HT interlayer enhancing energy level alignment and carrier transport	FTO/PEDOT:PSS/(Cs_0.02_(FA_0.9_ DEA_0.1_)_0.98_)_0.98_EDA_0.01_SnI_3_/EDA/PCBM:P3HT:ICBA/C_60_/BCP/Ag	GeI_2_, SnF_2_	25.83	0.753	75.3	14.64	154
SnT_2_ additive reducing defects and deepening the energy level of perovskite	FTO/PEDOT:PSS/(Cs_0.02_(FA_0.9_ DEA_0.1_)_0.98_)_0.98_EDA_0.01_SnI_3_/EDA/PCBM:P3HT:ICBA/C_60_/BCP/Ag	SnT_2_, GeI_2_, SnF_2_	24.72	0.843	73.6	15.33	104
Multidentate fullerene derivative (FM5) interlayer between perovskite and ETM	ITO/PEDOT:PSS/PEA_0.15_FA_0.85_ SnI_3_/PCBM/BCP/Ag	SnF_2_	24.54	0.86	71.1	15.05	161
Fullerene derivative without mixed-isomer bisadducts as the ETM	ITO/PEDOT:PSS/PEA_0.15_(FA_0.87_ MA_0.13_)_0.85_SnI_3_/Bis-PC/BCP/Ag	SnF_2_	21.69	0.86	66	12.27	162
Open-cage fullerene derivative as the ETM	ITO/PEDOT:PSS/PEA_0.15_FA_0.85_ SnI_3_/OC/BCP/Ag	SnF_2_, NH_4_SCN	19.6	0.72	68	9.6	163
Fluorinated triple-acceptor polymer (P3) as the ETM	ITO/PEDOT:PSS/PEA_0.1_FA_0.9_SnI_3_/P3/BCP/Ag	AET, SnF_2_	19.79	0.99	82.0	16.06	165
Pyridyl-substituted fulleropyrrolidines (PPF4) additive to regulate crystallization kinetics	ITO/PEDOT:PSS/PEACl/PEA_0.15_ FA_0.85_SnI_3_/C_60_BB/PCBM/BCP/Ag	PPF4, SnF_2_	26.17	0.86	71.4	16.05	14
Metal chalcogenide ETMs	ITO/Sn(S_0.92_Se_0.08_)_2_/PEA_0.15_FA_0.85_SnI_2.85_Br_0.15_/PTAA/Ag	AET, SnF_2_	20.47	0.73	71.1	11.78	170
Ambipolar SnO_*x*_ as HTM and top protective layer	FTO/SnO_*x*_/(Cs_0.02_(FA_0.9_DEA_0.1_)_0.98_)_0.98_EDA_0.01_SnI_3_/EDA/PCBM:ICBA/BCP/Ag	GeI_2_, SnF_2_	24.09	0.78	75	14.09	183
Pyrrolopyrrole-based polymer as HTM	ITO/PPr-SBT-14/anilinium iodide/FASnI_3_/C_60_/BCP/Ag	EDAI_2_, SnF_2_	22.37	0.51	66.1	7.6	177
Dibenzocarbazole-based-monolayer modifying NiO_*x*_	ITO/NiO_*x*_ NPs/2PADBC/EDA_0.01_FA_0.98_SnI_3_/PEAI/C_60_/BCP/Ag	SnF_2_	23.23	0.825	74.1	14.19	197
Monolayer-HTM-modified NiO_*x*_	ITO/NiO_*x*_/MBP/PEA_0.1_FA_0.9_SnI_3_/ICBA/BCP/Ag	SnF_2_, NH_4_SCN	22.48	0.99	80.7	17.89	16

Despite this progress, Sn-based PSCs still have considerable room for improvement in photocurrent generation, operational stability, and scalable fabrication. In many high-performing devices, the absorber layer remains relatively thin, typically 200–300 nm, which limits light harvesting and thus the achievable *J*_SC_. Increasing *J*_SC_ will therefore be one of the most important routes toward further efficiency improvement. However, simply increasing the thickness of the Sn perovskite layer is not sufficient. Because Sn-based perovskites crystallize rapidly and are vulnerable to Sn^2+^ oxidation, thicker films can suffer from rough morphology, nonuniform crystallization, higher defect densities, and enhanced bulk recombination. Future processing strategies must therefore enable thick, compact, and low-defect Sn perovskite films while maintaining efficient vertical charge transport and suppressing Sn^4+^ formation.

To achieve this goal, further advances in crystallization control will be essential. DMSO-free solvent systems, intermediate-phase regulation, vacuum-assisted crystallization, and additive-assisted nucleation control are promising approaches for producing uniform Sn perovskite films. At the same time, thicker absorbers should be evaluated not only by morphology and initial efficiency, but also by carrier lifetime, defect density, energetic disorder, and depth-dependent chemical composition. In particular, understanding the vertical distribution of Sn^4+^ species, Sn vacancies, residual additives, and compositional heterogeneity will be important for designing thick absorbers that enhance light absorption without sacrificing *V*_OC_ or FF.

In parallel, the ETM and perovskite/ETM interface must be further optimized. Because Sn-based perovskites generally possess shallower conduction band levels than Pb-based analogues, conventional C_60_ can introduce unfavourable energy offsets, leading to interfacial voltage losses and non-radiative recombination. Fullerene derivatives such as ICBA and fullerene bisadducts, as well as polymer-based ETMs, have been developed to improve energy-level alignment and reduce interfacial recombination. However, these approaches still face important challenges, including limited device reproducibility and insufficient operational stability. Future ETM design should therefore move beyond simple energy-level tuning toward robust materials and interlayers that simultaneously suppress interfacial recombination and enable stable electron extraction, particularly as thicker absorber layers are pursued to increase *J*_SC_. For regular n-i-p devices, further progress will require metal-oxide ETMs with controlled oxidation states, low defect densities, and high electron selectivity, together with interfacial treatments that suppress Sn^2+^ oxidation and redox reactions. Compatible deposition methods are also needed to form compact, low-defect Sn perovskite films directly on these oxide surfaces.

Another important direction is the reduction of optical and interfacial losses at the hole-collecting side. Sn-based PSCs still frequently employ polymeric hole-transporting materials such as PEDOT:PSS, which are convenient but can introduce parasitic absorption, reflection losses, and interfacial instability. In contrast, monolayer hole-transporting materials are ultrathin, optically transparent, and capable of forming selective contacts. The recent record efficiency obtained through buried-interface engineering highlights that the hole-collecting interface is not merely a passive contact, but an active component that controls wetting, perovskite nucleation, film quality, energy-level alignment, and interfacial recombination.

For Sn-based PSCs, however, monolayer HTMs must be designed more carefully than for Pb-based analogues. They should combine high transparency, suitable energy-level alignment, efficient hole extraction, suppression of interfacial oxidation, and appropriate surface energy for uniform Sn perovskite growth. In this sense, monolayer HTMs should be regarded as molecular platforms for controlling the buried interface, rather than simply as thinner replacements for polymeric HTMs. Their integration with thick, high-quality Sn perovskite absorbers could provide an effective route to higher *J*_SC_ while preserving *V*_OC_ and FF.

Long-term durability also remains a central challenge. Degradation of Sn-based PSCs can arise from Sn^2+^ oxidation, ion migration, interfacial redox reactions, and moisture or oxygen ingress. Although recent results have demonstrated encouraging operational stability, future studies should employ more systematic stress tests, including maximum-power-point tracking, thermal stress, damp-heat exposure, light/dark cycling, and combined stress conditions. Importantly, stability should be considered from the beginning of device design: additives, passivation molecules, and interfacial layers that improve initial efficiency must also remain chemically and electronically stable during long-term operation.

Finally, scale-up from small-area cells to large-area devices and modules will be critical. The recent demonstration of 14.4% efficiency for 1 cm^2^ Sn-based PSCs is an important step, but the efficiency gap between small-area and large-area devices indicates that scalable fabrication remains a major bottleneck. Large-area Sn perovskite films are particularly challenging because local variations in solvent evaporation, nucleation, additive distribution, and substrate wetting can lead to pinholes, thickness gradients, shunting pathways, and spatially heterogeneous recombination. Encouragingly, scalable and substrate-tolerant fabrication strategies are beginning to emerge, including a two-step blade-coating process^205^ and an antisolvent- and DMSO-free vacuum-quenching method that enabled a 21.6 cm^2^ module.^64^ These studies highlight that scalable Sn PSCs require precise control over intermediate formation, solvent removal, substrate wetting, and crystallization kinetics, rather than simply increasing the coating area. Future work should therefore place greater emphasis on scalable deposition methods such as blade coating, slot-die coating, bar coating, spray coating, vacuum-assisted deposition, and hybrid solution/vapour processes.

Overall, the field of Sn-based PSCs is entering a new stage. Suppressing Sn^2+^ oxidation and reducing defect densities will remain fundamental, but future progress will require integrated device design that simultaneously addresses light harvesting, interfacial selectivity, operational stability, and scalable fabrication. We believe that the next major advance in Sn-based PSCs will come from combining thick, low-defect absorbers with transparent and chemically benign charge-selective contacts, robust encapsulation, and reproducible large-area processing. This transition from high-efficiency small-area devices to stable and scalable Pb-free photovoltaics will define the next phase of Sn-based perovskite solar cell research.

## Author contributions

T. N. and C.-Y. C. wrote the original draft of the manuscript. All authors commented on the manuscript.

## Conflicts of interest

A. W. is co-founder and chief advisor (science & technology) of EneCoat Technologies, Co., Ltd.

## Data Availability

No primary research results, software or code have been included and no new data were generated as part of this review.
